# Author Correction: Large-scale plasma proteomics comparisons through genetics and disease associations

**DOI:** 10.1038/s41586-024-07549-z

**Published:** 2024-05-22

**Authors:** Grimur Hjorleifsson Eldjarn, Egil Ferkingstad, Sigrun H. Lund, Hannes Helgason, Olafur Th. Magnusson, Kristbjorg Gunnarsdottir, Thorunn A. Olafsdottir, Bjarni V. Halldorsson, Pall I. Olason, Florian Zink, Sigurjon A. Gudjonsson, Gardar Sveinbjornsson, Magnus I. Magnusson, Agnar Helgason, Asmundur Oddsson, Gisli H. Halldorsson, Magnus K. Magnusson, Saedis Saevarsdottir, Thjodbjorg Eiriksdottir, Gisli Masson, Hreinn Stefansson, Ingileif Jonsdottir, Hilma Holm, Thorunn Rafnar, Pall Melsted, Jona Saemundsdottir, Gudmundur L. Norddahl, Gudmar Thorleifsson, Magnus O. Ulfarsson, Daniel F. Gudbjartsson, Unnur Thorsteinsdottir, Patrick Sulem, Kari Stefansson

**Affiliations:** 1 deCODE Genetics/Amgen, Reykjavik, Iceland; 2 School of Engineering and Natural Sciences, University of Iceland, Reykjavik, Iceland; 3 School of Technology, Reykjavik University, Reykjavik, Iceland; 4 Department of Anthropology, University of Iceland, Reykjavik, Iceland; 5 Faculty of Medicine, School of Health Sciences, University of Iceland, Reykjavik, Iceland; 6 Faculty of Electrical and Computer Engineering, University of Iceland, Reykjavik, Iceland

**Keywords:** Quantitative trait, Proteomic analysis, Genome-wide association studies, Proteomics, Population genetics

Correction to: *Nature* 10.1038/s41586-023-06563-x Published online 4 October 2023

Due to an error in data analysis, several different samples from the same individual in the Icelandic data set measured using SomaScan were treated as repeated measurements of the same sample. This affects the computation of the coefficient of variation (CV) of repeated measurements of the same sample on the SomaScan platform, leading to an artificially high CV.

Furthermore, as the difference in distribution of protein levels between platforms may be at least partly due to population differences, we have removed attempts to evaluate the CV of repeated measurements relative to the CV of the assay in the population (CV ratio).

Both issues affect the conclusion of Olink assays being more precise than SomaScan assays, as using only the repeated measurements and computing the CV of repeated measurements of the same sample instead of the CV ratio, Olink assays exhibit higher CV than SomaScan assays, indicating higher precision of the SomaScan assays.

Finally, we have identified a couple of errors in our processing of raw measurements: while the Olink measurements were transformed with a base-2 logarithm, the SomaScan measurements were transformed with the natural logarithm, and an incorrect scaling factor was used to compute the MAD estimate of s.d. While these errors do not affect the conclusions, Extended Data Fig. 2 and Supplementary Tables 1, 2, 4 and 9 have been updated to reflect this.

As a consequence of the first two issues described, the section *Comparison of precision* has changed considerably (see the Supplementary Information for a list of edits and original article for comparison).

### Supplementary information

List of edits and original, uncorrected article

