# Supplementary material for: Large-scale plasma proteomics comparisons through genetics and disease associations
Source: Nature. 2023 Oct 4;622(7982):348–58. doi: 10.1038/s41586-023-06563-x (PMC10567571; doi:10.1038/s41586-023-06563-x)
Supplement: Supplementary file 1 — Supplementary Notes 1–20, Supplementary Tables 3–5, 7–11, 13 and 34, Supplementary Figs 1–9 and a guide to the annotation of columns found within Supplementary Tables 1–2, 6, 12, 14–33 and 35–40 (tables supplied separately). [file 41586_2023_6563_MOESM1_ESM.pdf]

nature portfolio

https://doi.org/10.1038/s41586-023-06563-x

Supplementary information

Large-scale plasma proteomics comparisons through genetics and disease associations

In the format provided by the authors and unedited

Supplementary Notes

SN1. Comparison of CV from repeated samples and control samples

Both the Olink and SomaScan platforms include repeated measurements of control samples for quality control purposes. Prior research using CV of such control samples as a measure of precision has demonstrated lower CV for assays on a previous version of the SomaScan platform (1.2K) than on the Olink Explore 1536 platform[1–3]. We observe similar results in our data: on control samples, the CV of SomaScan assays is on average smaller than the CV of Olink assays (Supplementary Table ST6)[1].

SN2. Comparison of batch effects between Olink in UK Biobank and SomaScan in Iceland

Both Olink and SomaScan use repeated measurements of control samples, specific to the platform, for quality control purposes. Computing the variation in the measurements of the control samples using either pairs of measurements from the same plate or pairs from different plates allows us to evaluate to what extent the variation is inherent to the assay itself and to what extent due to variability between plates, and how those variations relate to the total variation of the assay. We evaluated the variation in measurement of control samples in the Icelandic data set (measured by SomaLogic) and the UKB data set (measured by Olink) using the MAD to estimate the standard deviation, calculating the variance as $\sigma^2 = (1.4825 * \mathrm{MAD})^2$. Assuming that the variance on the control samples inherent in the assay σ_a^2 and the variance due to batch effect σ_p^2 are not correlated, we can decompose the total variance of the control sample measurements as $\sigma_M^2 = \sigma_p^2 + \sigma_a^2$.

In order to determine the impact of the plate effect on measurement results, the plate effect must be considered in relation to the total variance of the assay, σ^2. We can then evaluate how much of the total variance is due to the variability inherent in the assay (σ_a^2/σ^2) and how much due to batch effects ($\frac{\sigma_M^2-\sigma_a^2}{\sigma^2}$).

We can estimate σ_a^2 by using the difference between the two control samples measured on each plate, while we can estimate σ_M^2 by considering the control samples without regards to plate, and σ^2 from all values for the assay in question.

The fraction of assay variance explained by the variability inherent in the assay was 0.23 on the Olink platform and 0.10 on the SomaScan platform, while the fraction of variance explained by batch effects was 0.06 on Olink and 0.02 on SomaScan (Supplementary Figure SF9).

The discrepancy between the assay precision evaluated using the CV of duplicate measurements of samples and the variation of measurements of control samples may be in part due to the use of different control samples between the platforms.

SN3. Inter-class correlation:

The median correlation of the different assays for the two measurements of the same sample was lower for the Olink platform than the SomaScan platform (Spearman r=0.78 vs 0.99, respectively, Mann-Whitney p=6.6x10^{-130}). Both platforms emphasize the relative quantification within each assay, and neither of the platforms makes a claim towards the comparability of inter-class measurements. In fact, the normalization process for the Olink data shifts the median measurement for each assay to 0, and the difference between the levels of any two proteins on the Olink assay are therefore purely due to the position of the individual sample within the distribution of the assay. Conversely, for the SomaScan platform, the levels measured by two different assays can have vastly different medians, to the extent that the variation within each assay is dominated by the difference in medians. This ensures that for such SomaScan assays, any two measurements done with the assay with the higher median will always be higher than any two measurements done with the other, leading to stronger inter-class correlation between them. When adjusting for differences in data processing by shifting the median for each assay on each platform to 0, the median correlation remained lower for Olink than for SomaScan, although the difference was smaller (Spearman r=0.78 vs 0.83, respectively, Mann-Whitney p=6.4x10^{-7}; Supplementary Figure SF5).

SN4. The impact of SomaScan SMP normalization

As a normalization step, SomaScan scales each sample to conform to a reference population. This is done by calculating a scaling factor for each assay relative to a reference sample, averaging this scaling factor over all assays in the same dilution group, and applying the resulting scaling factor to all assays in the dilution group. While this can serve to reduce variation between samples, this may not always be desirable, as this has the effect of potentially removing large-scale variation in protein levels, affecting multiple proteins. Some studies therefore forego this normalization[4]. Here, we briefly note how the SMP normalization affects the present analysis.

SMP normalization reduces the median CV of the SomaScan assays (SMP: 9.9%, no SMP: 16.2%, Wilcoxon signed-rank $p<10^{-300}$).

The SMP normalization significantly reduces the median correlation between matching assays from the SomaScan and Olink platforms (SMP: r=0.33, no SMP: r=0.39, Mann-Whitney $p=1\times10^{-11}$), as well as the median correlation between random pairs of assays from the different platforms (SMP: -r=-0.01, no SMP: r=0.08, Mann-Whitney $p<1\times10^{-300}$). SMP normalization also had a large effect on median within-platform correlation (SMP: -0.08, no SMP: 0.42, Mann-Whitney $p<1\times10^{-300}$).

Using non-normalized data, the levels of 79% of SomaScan assays associated with age and 58% associated with sex (Supplementary Table ST14). The SomaScan normalization factors were associated with age and sex and affected the association with the normalized data accordingly (Supplementary Table ST14). The correlations between age and sex effects were less consistent between Olink and SomaScan (Spearman correlation=0.53 for age and 0.57 for sex).

In Iceland, we found 355,938 protein-phenotype associations ($p<1.0\times10^{-5}$) in the non-normalized SomaScan data (Supplementary Table ST16) compared to 218,503 associations for the SMP-normalized SomaScan data. Of these, 173,912 were significant for both normalization methods; 49% and 80% of the associations for the non-normalized and normalized data, respectively. However, 18% of the protein phenotype associations that were significant for both normalization methods had a discordant direction of effects. The direction of association was more likely to be concordant between non-normalized and normalized levels for positive associations (94%) than negative ones (78%). The discordant associations were mostly with phenotypes that are likely to associate with overall protein concentration, e.g., body mass index, blood cell counts, and markers of inflammation, liver function, and kidney function.

We identified 1,889 and 16,778 sentinel *cis* and *trans* pQTLs using the non-normalized SomaScan data, respectively, and 2,120 and 22,616 sentinel *cis* and *trans* pQTLS using the SMP normalized data (Table 1). Most non-normalized sentinel *cis* and *trans* pQTLs corresponded ($r^2>0.8$) to a sentinel normalized pQTL: 89% and 74%, respectively (Supplementary Tables ST19 and ST20).

In Iceland, we performed 164 billion tests (4,907 assays × 33.5 million variants), detecting 18,667 and 24,736 sentinel pQTLs for the non-normalized and normalized analyses, respectively, corresponding to estimated false discovery rates of 1.6% and 1.2%.

Using non-normalized SomaScan data we found 38 pQTLs associating with more than 50 proteins. compared with 35 using the SMP-normalized data. The pleiotropic SomaScan pQTLs each had at least 27 protein associations for either normalization method, indicating that the normalization does not create or remove pleiotropic pQTLs. However, the pattern of association was substantially altered by normalization for some pleiotropic variants. For example, the del12 variant in *ASGR1* went from affecting 556 proteins positively and no proteins negatively in the non-normalized data, to affecting 288 proteins positively and 331 proteins negatively in the normalized data.

We identified 1,889 and 16,778 sentinel *cis* and *trans* pQTLs using the non-normalized SomaScan data, respectively, and 2,120 and 22,616 sentinel *cis* and *trans* pQTLs using the normalized data (Table 1). The normalization suggested by SomaLogic leads to the detection of more pQTLs. However, this normalization correlates associates with many processes that affect the levels of multiple proteins.

This is reflected by discordant correlations between protein levels and phenotypes related to BMI, inflammation, blood cell counts, and kidney and liver function and by the drastic effects of normalization on pleiotropic variants like the ASGR1 variant which associates with higher levels of many proteins in non-normalized data. Notably, the normalization decreases the correlation between Olink and SomaScan assays, probably because it is adjusting out some of the biological variability as previously described. However, the power to detect protein associations with sequence variants and phenotypes that are not pleiotropic tends to increase following normalization.

SN5. Between-platform correlation of protein levels for shared targets

As the Olink NPX normalization leads to the mean level of each protein being 0 across the data set, there is no correlation on median protein levels between platforms, and neither can the variation in protein level IQR be explained by the intensity on the Olink platform. However, the protein level IQR was correlated between the two platforms (Spearman correlation 0.12, p=1.70×10^{-8}), and on both platforms, higher IQR was correlated with better correspondence with the matching assay (Spearman correlation for Olink assay IQR 0.28, p=4.84×10^{-39}; for SomaScan assay IQR 0.52, p=1.57×10^{-144}, Supplementary Figure SF2). This indicates that if either of a pair of assays has a low variation in levels, the correlation with the corresponding assay tends to be low.

Olink's data processing shifts the mean of the measurements for each protein to 0. Therefore, the comparison of the correlation of assay intensity with the correlation between the assays is not informative.

SN6. Between-platform ranking of assays by correlation of levels

To assess whether the matching assays on the two platforms are measuring the same proteins, we compared the levels of all pairs of targeted proteins between platforms. For each assay, we ranked the assays on the other platform by their correlation with the given assay (Supplementary Figure SF3). For Olink Explore assays, the matching SomaScan assay (with the highest correlation, if more than one was matching) ranked in the top 1% of all assays in 874 of 1,864 cases. Conversely, for SomaScan assays, the matching Olink Explore assay (with the highest correlation, if more than one was matching) ranked in the top 1% of all assays in 968 of 1,994 cases.

SN7. Association of ADM with heart failure

In addition to BNP, we noted an association with levels of protein encoded by the Adrenomedullin gene (*ADM*) which encodes pro-adrenomedullin which is cleaved into two peptides, ADM (adrenomedullin) and PAMP (proadrenomedullin N-terminal 20 peptide). Two assays on SomaScan (7922-5 and 14115-34) target protein products encoded by *ADM* and the correlation between them is

low (r=-0.12). A protein product encoded by *ADM* is also targeted on the Olink platform. One of the SomaScan assays correlates positively with the Olink assay (7922-5; r=0.42) whereas the other does not (14115-34; r=-0.24). Furthermore, both the Olink assay and the positively correlated SomaScan assay (7922-5) have a *cis* pQTL, while the negatively correlated SomaScan assay (14115-34) does not. Both the Olink assay and the positively correlated SomaScan assay (7922-5) associate with heart failure. Levels of products encoded by *ADM* have a strong association with heart failure on Olink (OR=1.85 per SD, p=6.69×10^{-72}), but a weaker association on the SomaScan 7922-5 assay (OR=1.23 per SD, p=2.69×10^{-19}). This is consistent with reports of elevated circulating ADM in heart failure[5]. In contrast, the negatively correlated SomaScan assay (14115-34) is negatively associated with heart failure (OR=0.81 per SD, p=2.49×10^{-23}). This underlines the need for detailed characterization of the proteoforms measured.

SN8. Our pQTL analyses using Olink in UK Biobank

In the UKB-BI Olink data, for 19% of the 26,926 sentinel pQTL associations, a secondary variant in the same region associated with the same protein based on conditional analysis (8,640 secondary associations in *cis* and 5,592 in *trans*). Combined, we found a total of 41,158 sentinel and secondary pQTL associations.

Most Olink sentinel *cis* pQTL associations had secondary pQTL associations (1,702 of 2,102; 81%), in contrast to only 3,340 (13%) of the 24,824 sentinel *trans* pQTL associations.

Of the Olink pQTLs, 6,530 (73%) associated with the level of a single protein, 2,154 (24%) with levels of two to nine proteins, and 320 (4%) with levels of ten or more proteins. We refer to pQTLs that were associated with levels of fewer than ten proteins as *specific* and the rest as *nonspecific*. Specific variants were associated with 2,416 (82%) of the Olink assays.

Of the 2,931 Olink assays for which GWAS analysis was performed, 3% had only *cis* pQTLs, 18% had only *trans* pQTLs and 68% had both *cis* and *trans* pQTLs. Furthermore, 10% had no pQTLs, whereas 9% had a single pQTL and 82% had two or more pQTLs.

SN9. Subsampling and accounting for the different number of variants tested

We performed a power analysis showing that reducing the sample size in the UK Biobank to be equal to the sample size in Iceland (n=35,892) would be expected to result in the detection of 3% fewer sentinel *cis* pQTL and 34% fewer *trans* pQTL associations: if the sample size in UKB were 35,892, for the overall set of assays, we would expect to detect 2,021 of the sentinel *cis* associations (96% of the 2,102 detected in the full set of individuals), and 16,399 of the sentinel *trans* associations (66% of the 24,824). When restricting to assays measuring proteins targeted by both platforms, we would expect

to detect 1,423 of the sentinel *cis* associations (97% of 1,468), and 12,315 of the sentinel *trans* associations (66% of 18,578). The majority of the comparisons between platforms that we have performed on pQTL focused on *cis* pQTLs. Using a smaller set (36K) in UK-BI using Olink would therefore not impact our conclusions since the fraction of assays with *cis* pQTLs in UKB on the overlapping set would go from 80% to 78% with Olink instead of 60% with SomaScan.

In Iceland, we performed 164 billion tests (4,907 assays × 33.5 million variants), detecting 24,736 sentinel pQTLs, corresponding to an estimated false discovery rate of 1.2%. For the UKB-BI data set, taking into account 169 billion tests (2,931 assays × 57.7 million variants), detecting 26,926 sentinel pQTL associations corresponds to an estimated false discovery rate of 1.2%.

Correcting for the number of variants tested in UKB (57.7 million) would result in a multiple testing correction threshold in UKB of 8.7×10^{-10} instead of 1.8×10^{-9}. A total of 153 (1%) of the *cis* pQTLs have significance between those two thresholds and 1,608 (5%) of the *trans* pQTLs. This does not affect the conclusions of the comparison between the platforms.

SN10. Replication of UKB-BI pQTLs in Icelandic Olink data

We tested the sentinel pQTLs we discovered using the UKB-BI data set for association using Olink data from 1,514 Icelanders and found 74% and 19% of the *cis* and *trans* pQTL associations to be nominally significant (p < 0.05) and with consistent directions (Supplementary Table ST35). In comparison, a power analysis resulted in expected proportions of replicated associations of 85% and 35% for *cis* and *trans* pQTL associations, respectively. The ratio between expected and observed replication was 0.87 and 0.54 for *cis* and *trans* pQTLs, respectively, and while the power analysis represents a best-case scenario, the replication rate may also be impacted by differences in population and sample handling and processing (Supplementary Table ST13).

SN11. *Cis* pQTLs in high LD with a PAV and not *cis* eQTL

In general, a *cis* pQTL would be expected to be equally likely to correlate with increased levels of a protein as decreased. Therefore, the average effect of *cis* pQTLs would be expected to be close to zero. However, if the pQTL is due to epitope effects, the alternate allele would be more likely to associate with decreased levels of the protein as measured by the assay. As we have previously observed for SomaScan, the effect of the alternative allele of *cis* pQTLs not in high LD with a PAV is on average close to zero[4]. However, when a PAV is in high LD with a *cis* pQTL, we observe a negative effect for the alternative allele and the negative effect tends to be even stronger when no *cis* eQTL is in high LD. When the PAV in high LD is annotated as high impact (stop gained, frameshift, splice donor or acceptor), the negative effect is consistent with expected nonsense-mediated decay, in particular

when supported by *cis* eQTL. However, when the PAV in high LD is annotated as moderate impact (missense, inframe indel, or splice region), the negative effect we observe for the alternative allele is consistent with epitope effect.

SN12. pQTL replication between the Olink and SomaScan platforms

Replication of pQTLs detected using SomaScan has been attempted using Olink Target for a limited set of proteins (n=90 and 163)[6]. In those studies, 65% (81% for *cis* and 52% for *trans*) and 67% (93% for *cis* and 64% for *trans*) of pQTLs identified in the Interval and deCODE studies replicated, respectively[4,6,7]. Reciprocally, 64% (75% for *cis* and 61% for *trans*) of the pQTLs identified using Olink Target were replicated using SomaScan (n=163 proteins)[4,6]. A recent study involved a larger number of proteins targeted by both platforms (n=871) but was limited by the low number of participants measured using Olink Target (n=485)[8].

In addition to the correspondence of pQTLs, for comparison with previous work in the field, we checked the replication of pQTLs detected on each of the platforms on the other platform[7,8]. Of the pQTLs we identified using Olink UKB-BI, we nominally ($p < 0.05$) replicated 70% of sentinel *cis* pQTLs and 54% of sentinel *trans* pQTLs in the Icelandic SomaScan data (Supplementary Table ST30). Conversely, we replicated 84% of sentinel *cis* pQTLs and 54% of sentinel *trans* pQTLs detected using SomaScan data in the UKB-BI Olink data (Supplementary Table ST37). Thus, the replication rate using Olink for *cis* pQTLs detected using SomaScan was somewhat higher than the replication rate using SomaScan for *cis* pQTLs detected using Olink.

When attempting to replicate sentinel Olink pQTLs in UKB-BI using Icelandic SomaScan data, the correlation between effect estimates was similar for *cis* pQTLs (r=0.51; 95% CI: 0.47-0.55) and *trans* pQTLs (r=0.48; 95% CI: 0.47-0.49) (Extended Data Figure EF7a, b). However, the correlation between effects was substantially higher for *cis* pQTLs not in high LD with a PAV (r=0.63; 95% CI: 0.58-0.66) than for *cis* pQTLs in high LD with a PAV (r=0.42; 95% CI: 0.33-0.50) (Extended Data Figure EF7c). For pQTLs detected using SomaScan, the correlation with effect estimates using Olink was somewhat higher for *cis* pQTLs (r=0.48; 95% CI: 0.43-0.53) than for *trans* pQTLs (r=0.41; 95% CI: 0.39-0.42) (Extended Data Figure EF7d, e). As in the other direction of replication, the correlation was substantially higher for *cis* pQTLs not in high LD with a PAV (r=0.74; 95% CI: 0.70-0.77) than for *cis* pQTLs in high LD with a PAV (r=0.31; 95% CI: 0.21-0.40) (Extended Data Figure EF7f).

Assays targeting proteins with *cis* pQTLs that did not replicate between platforms had lower correlation between measured protein levels than assays for proteins with replicating *cis* pQTLs (Extended Data Figure EF7 a, b, d, e; Supplementary Tables ST30, ST31, and ST6). For *cis* pQTLs detected using the SomaScan v4 platform and replicated on the Olink Explore platform, this is based on 894 replicating

and 164 non-replicating pQTLs, while for *cis* pQTLs detected using the Olink Explore platform and replicated using the SomaScan v4 platform, this is based on 871 replicating and 375 non-replicating pQTLs.

To investigate if the observed difference in replication between the cohorts might be caused by differences in LD structure, we computed for both cohorts the r2 in the source population between each reported sentinel pQTL variant and the variants in the credible sets for the pQTL, and compared the r2 for variants in high LD (r2 > 0.8) with their r2 in the replication cohort.

Of the sentinel pQTLs detected in the Icelandic data set, 1,527 out of 9,611 variants (16%) did not map to the UKB-BI data set, while of the sentinel pQTLs detected in the UKB-BI data set, 3,864 out of 13,641 variants (28%) did not map to the Icelandic data set. In both cases, rare variants (MAF < 1%) were less likely to map than others, with 444 out of 1,421 (31%) variants detected in Iceland and 616 out of 1,074 (57%) variants detected in UKB-BI failing to map to UKB-BI and Iceland, respectively.

Of the variants in high LD with the reported sentinel variant in the Icelandic cohort (r2 > 0.8), 92% were also in high LD in the UKB-BI cohort, while 89% of the sentinel variants in the UKB-BI cohort were also in high LD in the Icelandic cohort. A further 5% and 8% of the variants in high LD with the sentinel reported pQTLs from the Icelandic and UKB-BI data, respectively, had r2 between 0.6 and 0.8. Thus, for both cohorts, 97% of the variants in high LD with the sentinel reported pQTL had r2 > 0.6 in the replication cohort (Supplementary Figure SF7, Supplementary Tables ST32-ST34). It is therefore unlikely that the difference in replication rate between the platforms is because of difference in LD structure between the cohorts.

SN13. Replication of trans pQTLs conditional on cis pQTLs

For pQTLs detected in UKB-BI, subdividing signals into proteins having *cis* pQTLs on neither, only one, or both platforms resulted in 3%, 2%, and 13%, respectively, of signals having a sentinel corresponding *trans* pQTL in the Icelandic SomaScan data. Similarly, for pQTLs detected in the Icelandic SomaScan data, subdividing into proteins having *cis* pQTLs on neither, only one, or both platforms resulted in 3%, 4%, and 25%, respectively, of signals having a sentinel corresponding *trans* pQTL in the UKB-BI Olink data (Supplementary Table ST35).

SN14. Variants associating with a large number of proteins

The two variants associating with the largest number of proteins based on SomaScan but not on Olink are close to *CFH* and *SKIV2L*. The two variants associating with the largest number of proteins on Olink but not on SomaScan are close to *SLC22A4* and *GP6*. The variant at *GP6* is in high LD with a missense variant (Pro219Ser) in *GP6*, it is an eQTL and a pQTL for GP6, and associates with platelet volume[9] and

aggregation[10]. GP6 is the primary glycoprotein receptor that mediates collagen responses in platelets involved in thrombus formation and the difference in the associations between the platforms may reflect differences in plasma sample preparation and handling[4,11,12], as prolonged storage of blood samples may lead to platelet activation and subsequent secretion of proteins into plasma[11,13].

SN15. Concurrence between GWAS catalog LD with pQTL and credible set.

For sentinel pQTLs in the Olink UKB-BI data, there were 669,045 pairs where variants in high LD associated with levels of a particular protein and a phenotype in the GWAS catalog; 4,439 pairs in *cis* and 664,606 pairs in *trans*. Of those, 2,409 (54%) of the *cis* pairs and 529,604 (79%) of the *trans* pairs also have the GWAS catalog variant included in the credible set of the pQTL association.

For sentinel pQTLs in the Icelandic SomaScan data, there were 300,067 pairs where variants in high LD associated with levels of a particular protein and a phenotype in the GWAS catalog; 3,360 pairs in *cis* and 296,707 pairs in *trans*. Of those, 1,597 (48%) of the *cis* pairs and 196,836 (66%) of the *trans* pairs also had the GWAS catalog variant included in the credible set of the pQTL association.

SN16. Proteins targeted by both platforms, same *cis* pQTL for both platforms

The protein encoded by *SULT2A1* (bile salt sulfotransferase) is targeted by both Olink and SomaScan platforms and the *cis* pQTL from both platforms, rs212100 (p=2.2×10^{-410}; effect=-0.38 SD) and chr19:47871693 (non-normalized p=2.5×10^{-90}; effect=-0.21 SD, normalized p=6.5×10^{-122}; effect=-0.25 SD), are in high LD (r^2=1; MAF=16%). SULT2A1 levels are correlated between the platforms (r=0.48). We and others have reported association of the minor allele of rs212100 with less gallstone risk[14] and lower plasma levels of SULT2A1 using the SomaScan v4 platform[4,15]. The results here using the Olink Explore platform in UKB confer an independent replication. These variants are reported to be in high LD with the sentinel *SULT2A1* eQTL (Lung and Liver effect on RNA=-0.90 SD), but not with any PAV.

Similarly, we had reported relationship between a CHRDL2 *cis* pQTL and a colorectal cancer risk variant using SomaScan, implicating *CHRDL2* at the locus as the gene through which the effect is likely mediated in the absence of any associated eQTL or PAV[4]. Consistent with these results, the sentinel *cis* pQTL (rs11236228; MAF=4%) for CHRDL2 in Olink is in high LD (r^2=1) with the disease-associated variant (rs4944940) with a similar effect *(*p=2.9×10^{-374}; effect=-0.68 SD) to the one observed on SomaScan (effect=-0.62SD), thus providing an independent replication.

SN17. Comparison of pQTLs for IL34

The sentinel *cis* pQTL on Olink for IL34 is a stop-gained variant in *IL34* (rs4985556), located in the last exon (position 213 of 243 amino acids) and associates with greater Alzheimer's disease (AD) risk

(OR=1.08, $p=2\times10^{-8}$)[16]. The stop-gained variant associates with lower IL34 levels ($p=1.3\times10^{-3260}$; effect=-1.09 SD; MAF=12%) which is consistent with the predicted loss of function. No *cis* pQTLs were detected for IL34 on SomaScan, and the correlation between the levels of IL34 measured on the two platforms is low (r=0.04). IL34 promotes microglial proliferation and clearance of soluble amyloid-β (Aβ) and as such confers neuroprotection against the neurotoxicity of Aβ. It has been proposed that the stop-gained variant associating with less IL34 could increase AD risk by reducing the neuroprotective properties of IL34[16].

SN18. Proteins targeted by both platforms, different *cis* pQTL detected by the platforms

In some instances, *cis* pQTLs for a given protein are detected on both platforms but they have little correlation and only one of them correlates with a disease-associated variants. In the case of the CD58 (Lymphocyte function-associated antigen 3) protein we observed *cis* pQTLs on both the Olink Explore and SomaScan v4 platforms, however, the *cis* pQTLs were different. The sentinel *cis* pQTL on Olink Explore (rs10801908, MAF=12%) and SomaScan v4 (rs61789227; MAF=2.1%) have very different minor allele frequencies and are in low LD (r^2=0.04) (Extended Data Figure EF8). However, a secondary *cis* pQTL for CD58 on SomaScan v4 (rank 2, rs10754443; MAF=11%; effect=-0.10 SD, $p=3\times10^{-16}$) is in high LD (r^2=0.81) with the sentinel *cis* pQTL on Olink Explore. None of the *cis* pQTLs on the two platforms associate with a PAV. The correlation between the levels of CD58 measured on the two platforms was low (r=0.02). The sentinel *cis* pQTL (rs10801908) for CD58 on Olink Explore ($p=2.2\times10^{-147}$, effect=-0.26 SD) is reported to associate with 25% less risk of multiple sclerosis (MS)[17,18]. The minor allele of this variant associated with lower CD58 levels on Olink Explore and less MS risk. The glycoprotein CD58, also known as lymphocyte-function antigen 3 (LFA-3), is a costimulatory receptor distributed on a broad range of human tissues including cells of the immune system, making it relevant for autoimmune diseases, including MS[19]. Based on these results both platforms are likely targeting CD58 and the discrepancies observed could result from the two platforms targeting different CD58 isoforms, or multimeric versus monomeric forms.

SN19. Proteins targeted by either SomaScan or Olink

Olink Explore 3072 targets 2,923 proteins, of which 1,098 are not targeted by SomaScan v4, including ITGA11. The minor allele of Val433Met in *ITGA11* (rs2306022; MAF=9%) is the sentinel *cis* pQTL for ITGA11 ($p=3.3\times10^{-6217}$, effect=-0.37SD) and is reported to confer risk of Dupuytren contracture (OR=1.27, PP=0.99) and uterine fibroids (OR=1.12, PP=1.00)[20,21] (Supplementary Figure SF8). The fact that the variant which associates with ITGA11 levels is a PAV, and not a *cis* eQTL, raises the concern that the observed reduction in ITGA11 may be the result of an epitope effect. Validation of this thus needs to be performed using an alternative method.

Of the 4,719 proteins targeted on SomaScan v4, a total of 2,891 are not targeted on Olink Explore 3072. We have previously reported on instances of *cis* pQTLs on SomaScan for proteins that are not targeted by Olink and their relationship with disease-associated variants including GREM1 and colorectal cancer, ASIP and melanoma, and STAT3 and multiple sclerosis[4] (Supplementary Figure SF8).

SN20. Proportion of assays with *cis* pQTLs on the Olink and SomaScan platforms

A greater fraction of the Olink Expansion set of assays targets intracellular and less abundant proteins (undiluted) than of the 1536 set. Both these characteristics are associated with lower fraction of assays having a *cis* pQTL within each set of assays. Consequently, we observe a lower fraction of proteins with *cis* pQTLs for proteins targeted in the Expansion set of assays than by the initial 1536 set (56% vs 85%). Similarly, proteins targeted on a previous generation of the SomaScan platform (SomaScan 1.2K, Supplementary Table ST2) are more likely to have a pQTL in our data than proteins targeted on the SomaScan v4 platform and not the older version (40% vs 56%). Analyzing only the proteins targeted by both platforms, and considering separately the 1,109 and 739 proteins included in the 1536 and Expansion sets, the fraction of proteins with *cis* pQTLs was in both cases greater on Olink than on SomaScan (87% vs. 67% for 1536 and 67% vs. 51% for Expansion).

Supplementary tables

-	Olink Explore 3072	Olink Explore 1536	Olink Expansion	SomaScan (non-normalized)	SomaScan (normalized)
n_samples	1474	1474	1474	229	227
median cv	16.5334	13.9926	19.1373	16.2204	9.8715
q1	12.1503	10.9256	14.1360	13.5926	7.2613
mean	16.5334	13.9926	19.1373	16.2204	9.8715
q3	22.9726	20.2436	24.3455	20.8062	15.2698
iqr	10.8223	9.3180	10.2095	7.2137	8.0085
median overlap cv	14.9084	13.2008	18.5044	15.2557	9.5442
q1 overlap	11.5143	10.6146	13.4038	12.7109	7.2322
mean overlap	14.9084	13.2008	18.5044	15.2557	9.5442
q3 overlap	21.8905	18.6046	24.2382	19.5903	14.4378
iqr overlap	10.3762	7.9899	10.8344	6.8794	7.2056

Supplementary Table ST3: Distribution of CV for Olink and SomaScan assays

Quantile	0.1	0.25	0.5	0.75	0.9
Intra-assay					
SomaScan (1.2K)	0.02	0.03	0.03	0.04	0.06
SomaScan (all)	0.02	0.02	0.03	0.04	0.06
Olink (v1)	0.03	0.03	0.05	0.07	0.11
Olink (all)	0.03	0.04	0.06	0.11	0.17
Inter-assay					
SomaScan (1.2K)	0.04	0.05	0.06	0.08	0.12
SomaScan (all)	0.04	0.04	0.05	0.07	0.11
Olink (v1)	0.09	0.12	0.16	0.24	0.36
Olink (all)	0.10	0.14	0.20	0.31	0.50

Supplementary Table ST4: Assay CV, replicating the results of Katz *et al.*[1], showing higher precision of assays on the SomaScan platform. Comparing the assay CV directly does not take into account different scales of the platforms.

-	Olink vs somascan (non-normalized)	Olink vs somascan (normalized)	SomaScan normalized vs non-normalized
median_corr	0.3883	0.3255	0.737
min_corr	-0.2865	-0.3548	-0.377
max_corr	0.9356	0.9098	1
q1_corr	0.1124	0.0325	0.556
q3_corr	0.6254	0.5843	0.8651
iqr_corr	0.513	0.5518	0.3091
baseline_corr	0.0777	-0.0055	nan
q1_baseline_corr	0.0112	-0.0634	nan
q3_baseline_corr	0.2142	0.0611	nan

Supplementary Table ST5: Median Spearman correlation between assays on the Olink and SomaScan platforms using normalized (smp) and non-normalized (pc0) data.

	Olink dilution						
SomaScan dilution		1:1	1:10	1:100	1:1000	1:100000	total
	1:5	1050	300	107	27	4	1488
	1:200	119	110	116	60	17	422
	1:20000	3	4	11	36	59	113
	total	1172	414	234	123	80	2023

Supplementary Table ST7: Assay dilution groups for the Olink and SomaScan platforms.

			SomaScan dilution			
			1:5	1:200	1:20000	all
Olink dilution	1:1	count	1050	119	3	1172
		median normalized corr	0.157	0.273	0.105	0.161
		median non-normalized corr	0.240	0.301	0.197	0.243
	1:10	count	300	110	4	414
		median normalized corr	0.437	0.614	0.223	0.479
		median non-normalized corr	0.487	0.629	0.267	0.516
	1:100	count	107	116	11	234
		median normalized corr	0.335	0.594	0.480	0.516
		median non-normalized corr	0.382	0.615	0.494	0.540
	1:1000	count	27	60	36	123
		median normalized corr	0.165	0.544	0.576	0.511
		median non-normalized corr	0.334	0.603	0.562	0.545
	1:100000	Count	4	17	59	80
		Median normalized corr	0.138	0.359	0.451	0.426
		Median non-normalized corr	0.169	0.428	0.520	0.495
	all	count	1488	422	113	2023
		median normalized corr	0.208	0.528	0.483	0.325
		median non-normalized corr	0.297	0.569	0.520	0.388

Supplementary Table ST8: Median correlation between matching assays on the Olink and SomaScan platforms by dilution group

platform	dilution group	count	median cv	count (matching)	median cv (matching)
olink	1:1	1961	19.75546	1091	18.50443
	1:10	524	13.22939	384	12.81051
	1:100	257	11.23674	211	11.03967
	1:1000	127	11.60512	113	11.60512
	1:100000	72	11.11745	63	11.09153
somascan_non-normalized	1:5	4013	9.993957	1462	9.892588
	1:200	791	8.973037	419	8.045246
	1:20000	157	10.46488	113	10.27222
somascan_normalized	1:5	4013	16.79949	1462	16.17287
	1:200	791	13.58756	419	12.42079
	1:20000	157	14.79755	113	14.37824

Supplementary Table ST9: CV by dilution group for all assays on each platform and for the set of matching assays.

	All proteins according to HPA	SomaScan v4	SomaScan 1.2k	Olink Explore 3072	Olink Explore 1536
Intracellular	12159 (63%)	2425 (49%)	338 (30%)	1414 (46%)	562 (36%)
Membrane	5384 (28%)	1434 (29%)	336 (30%)	891 (29%)	532 (34%)
Secreted	1644 (9%)	1064 (22%)	443 (40%)	751 (24%)	463 (29%)

Supplementary Table ST10: The difference in protein location (according to Human Protein Atlas; HPA) between SomaScan and Olink.

		cis	no *cis*
50% of values	both below LOD	185	498
	one below LOD	94	83
	neither below LOD	1823	260

Supplementary Table ST11: Number of proteins with *cis* pQTL detected or not detected on Olink depending on if the median of the NPX values was below the limit of detection on the validation sets of samples from healthy individuals (n=24) and diseased individuals (n=48).

	UKB-BI	Iceland 36k	UVS	HERA
Country	UK	Iceland	Iceland	Iceland
Count	50104	36195	17560	18635
Donor age mean	57.9	56.3	58.4	54.4
Donor age std	8.1	16.9	17.1	16.5
BMI mean	27.3	27.3	26.5	28.2
BMI std	4.7	5	4.6	5.4
Sex proportion (female)	0.54	0.57	0.57	0.57
Bias	healthy	diseased	diseased	diseased
Sample age (years) mean*	13	10.2	16.7	4.2
Sample age (years) std	2.4	7.4	1.2	5.4
Plasma handling protocol	stand 30min, centrifuge, ship to processing center, aliquot	stand 30min, centrifuge, aliquot	stand 30min, centrifuge, aliquot	stand 30min, centrifuge, aliquot
Storage temperature	-80°C	-80°C	-80°C	-80°C
Freeze-thaw cycles	0	0	0	0
Delayed sample handling**	TRUE	FALSE	FALSE	FALSE

Supplementary Table ST13: Differences in the UKB-BI and Icelandic cohorts and study characteristics. Age is in years and the unit of BMI is kg/m^2. *assuming all SomaScan measurements performed in 2019 and all Olink measurements in 2022. **UKB samples were kept at 4°C until they were shipped to processing center for aliquoting, preferably within 24h[12]. Icelandic samples were aliquoted immediately following centrifuging.

r2 range	uk_to_is	is_to_uk
0.0-0.2	0.6%	1.6%
0.2-0.4	0.5%	0.5%
0.4-0.6	1.9%	1.1%
0.6-0.8	7.6%	5.2%
0.8-1.0	89.3%	91.7%

Supplementary Table ST34: LD between pQTL variants and credible set variants in UKB-BI and Iceland. For each pQTL we compute the LD in the source cohort between the pQTL and each variant in the credible set. For variants in high LD in the source cohort, the table shows the LD of the variants in the replication cohort. For both directions, 97% of the variant pairs had $r^2 > 0.6$.

Supplementary Figures

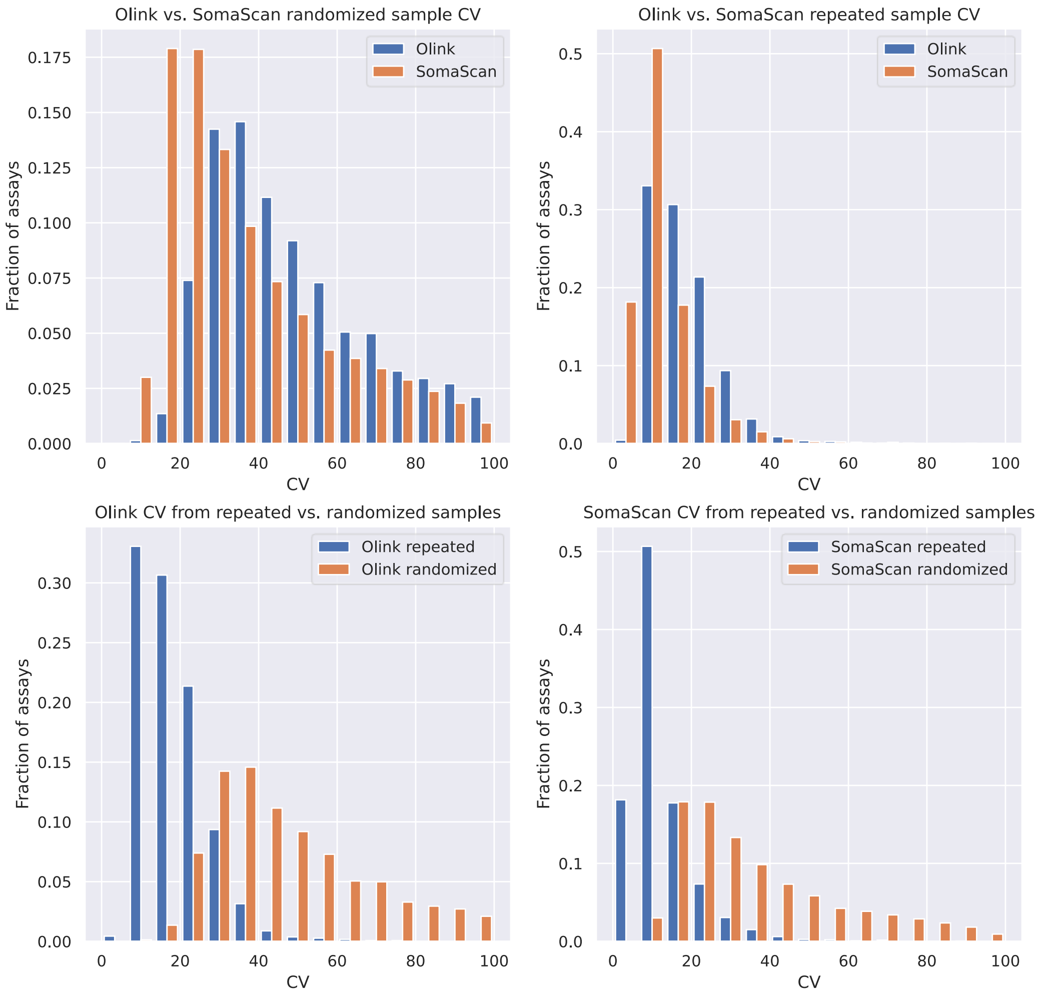

Supplementary Figure SF1: Top row: Comparison of assay CVs on the Olink and SomaScan platforms computed using random samples (left) and duplicate measurements of samples (right). Bottom row: Comparison of assay CVs computed using random samples and duplicate measurements of samples on the Olink (left) and SomaScan (right) platforms.

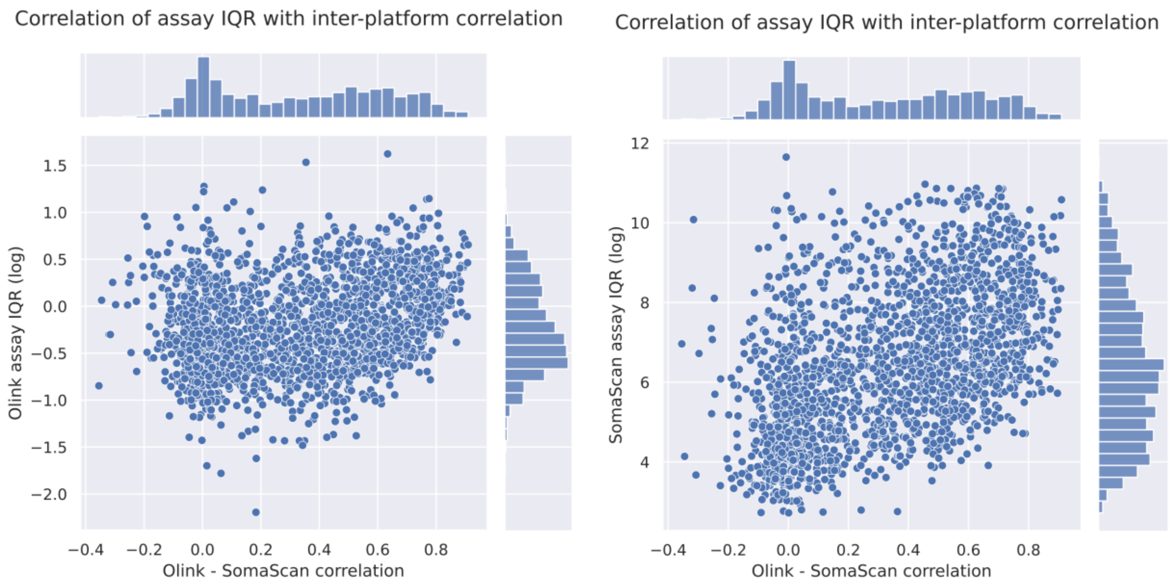

Supplementary Figure SF2: Relationship between assay IQR and inter-platform correlation. Assay IQR is correlated with inter-platform correlation, with higher assay IQR associating with higher inter-platform correlation.

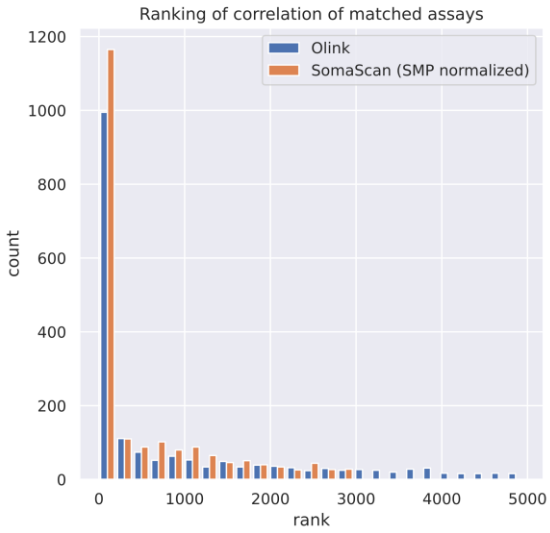

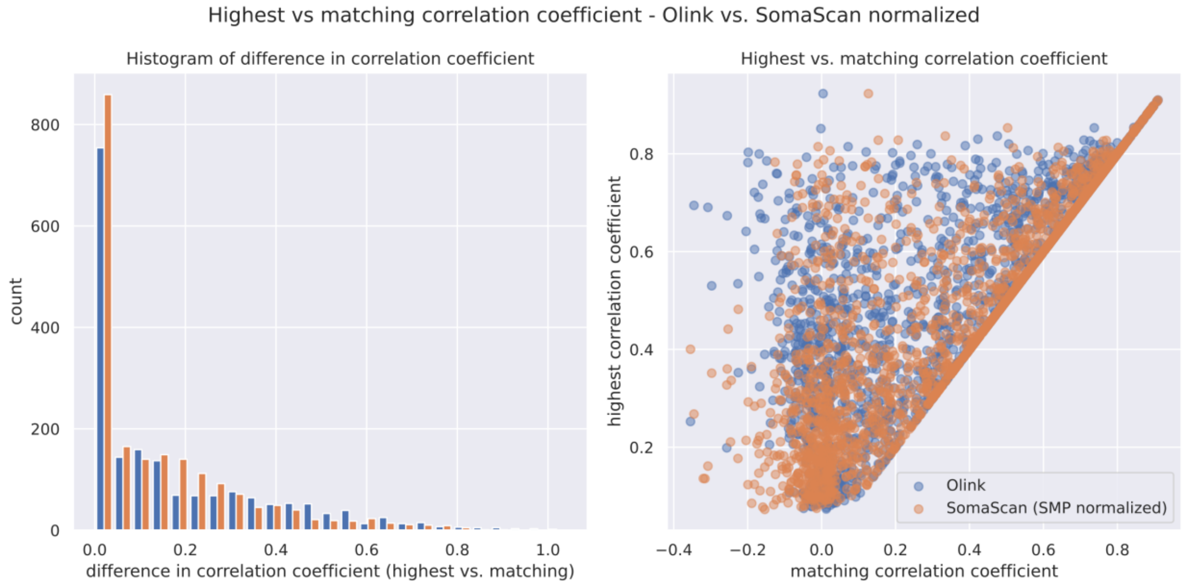

Supplementary Figure SF3: Top: Histogram of the rank of the matching assay compared to all possible pairs. For an assay on a given platform, the matching assay on the other platform is among the highest-ranked assays on that platform in a majority of cases.

Bottom: The difference between the highest correlation coefficient for an assay and the correlation coefficient for the matched assay. The histogram shows the distribution of the differences in correlation coefficient between the matching assay and the assay with the strongest correlation. The right-hand plot shows the distribution of the correlation coefficients. For assays on the diagonal, the matching assay is the assay showing the highest correlation in protein levels. For the majority of assays, the difference between the strongest correlation and the correlation with the matching assay is small.

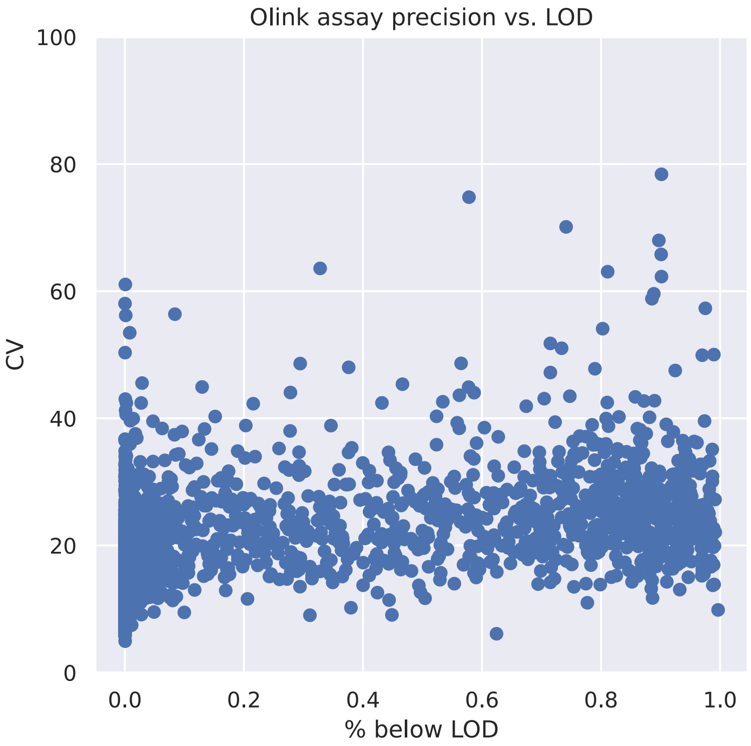

Supplementary Figure SF4: Correlation of CV to percentage of measurements below limit of detection for Olink assays. The Spearman correlation is 0.69, $p<10^{-300}$.

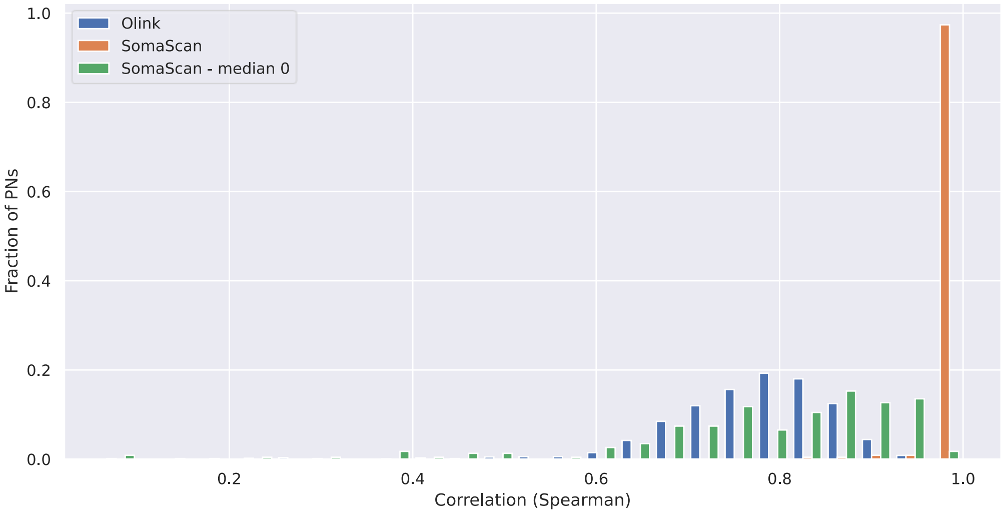

Supplementary Figure SF5: Histogram of intra-class correlation coefficients for Olink (UKB) and SomaScan (Iceland 36K) data sets. The correlation is computed for the paired samples in each data set. Shifting the values for each assay on the SomaScan platform to have a mean of 0, as is done for the Olink assays, greatly impacts the median correlation.

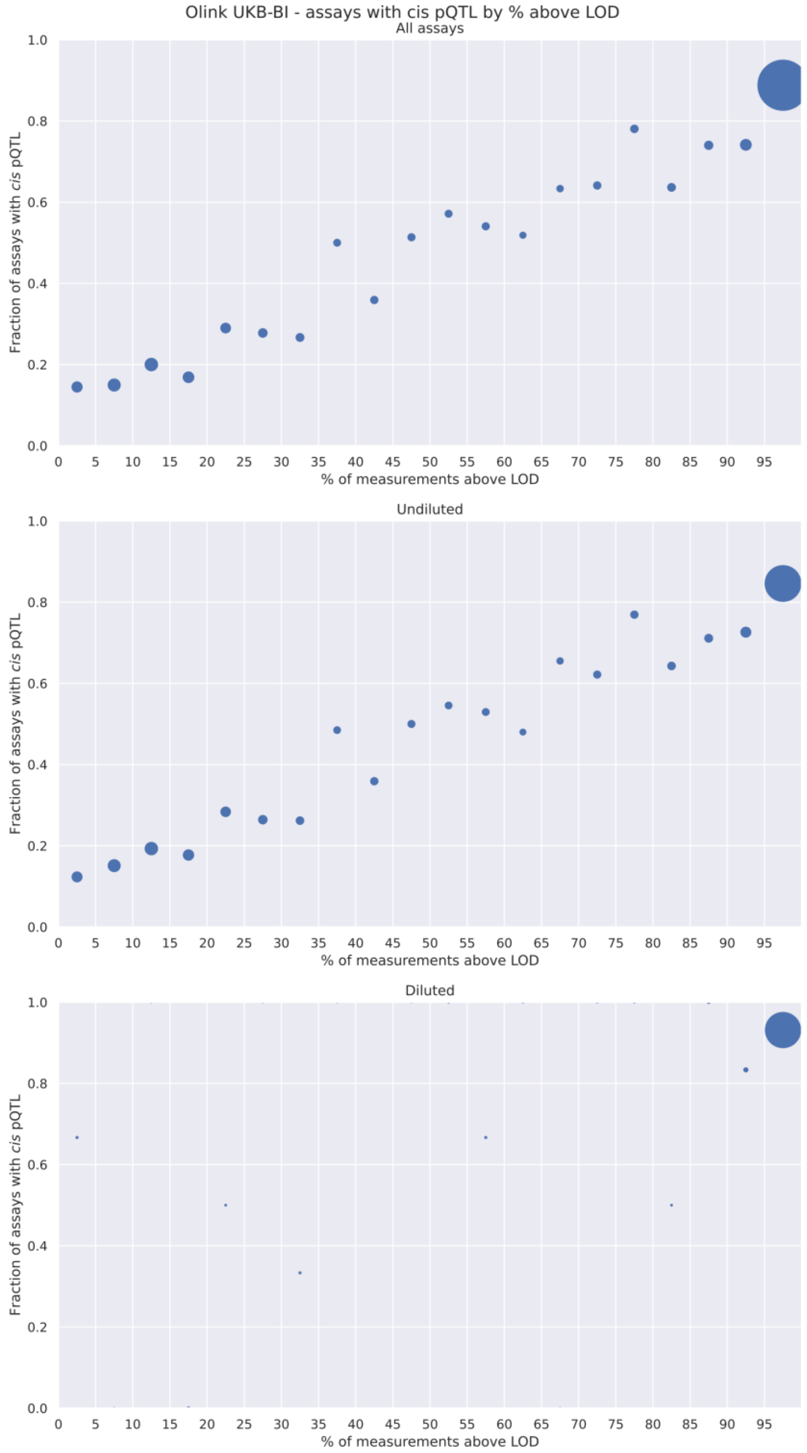

Supplementary Figure SF6: The fraction of assays with a *cis* pQTL compared to the percentage of measurements above LOD, increments of 5%. The area of the dots represents the number of assays in each bin.

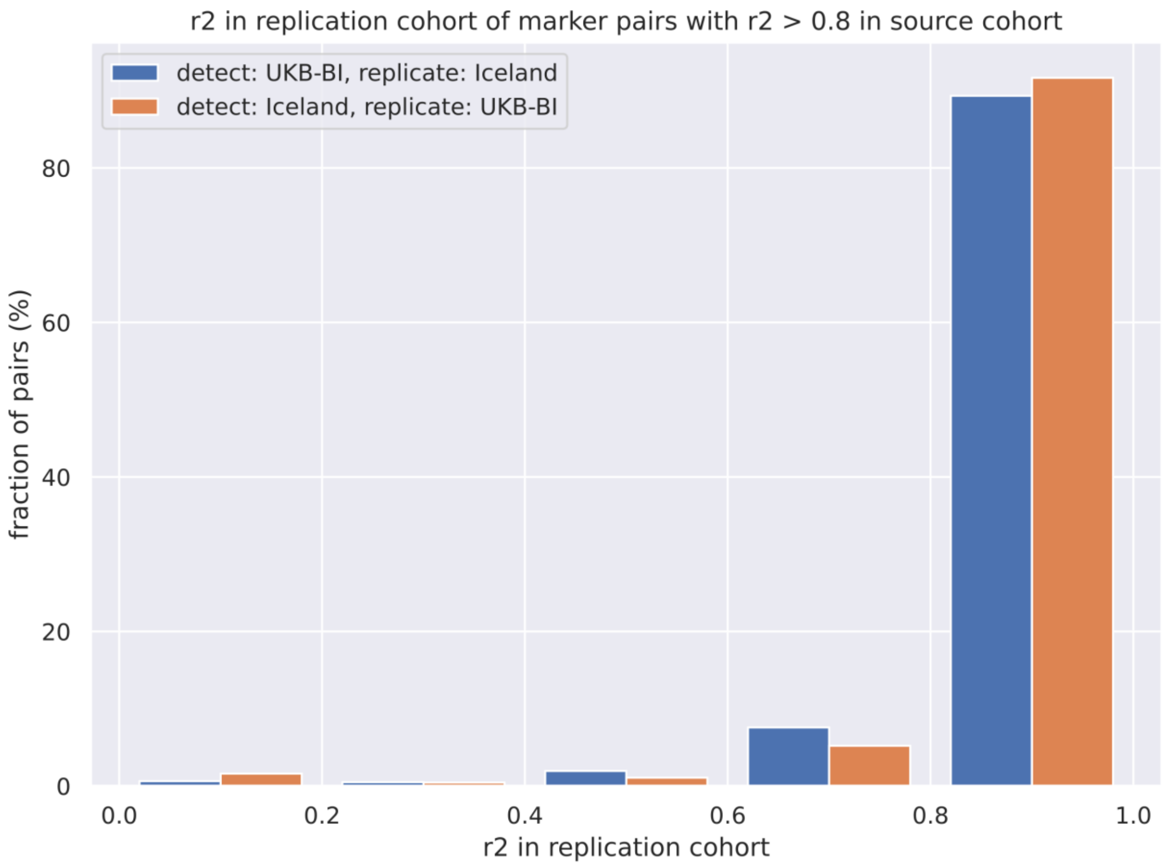

Supplementary Figure SF7: Histogram showing the r^2 in the replication cohort between pairs of a sentinel pQTL and a credible set variant from the source cohort having $r^2 > 0.8$ in the source cohort. For pQTLs detected in both data sets, 97% of the pairs have $r^2 > 0.6$, with $r^2 > 0.8$ in about 90% of cases (Supplementary Table ST34).

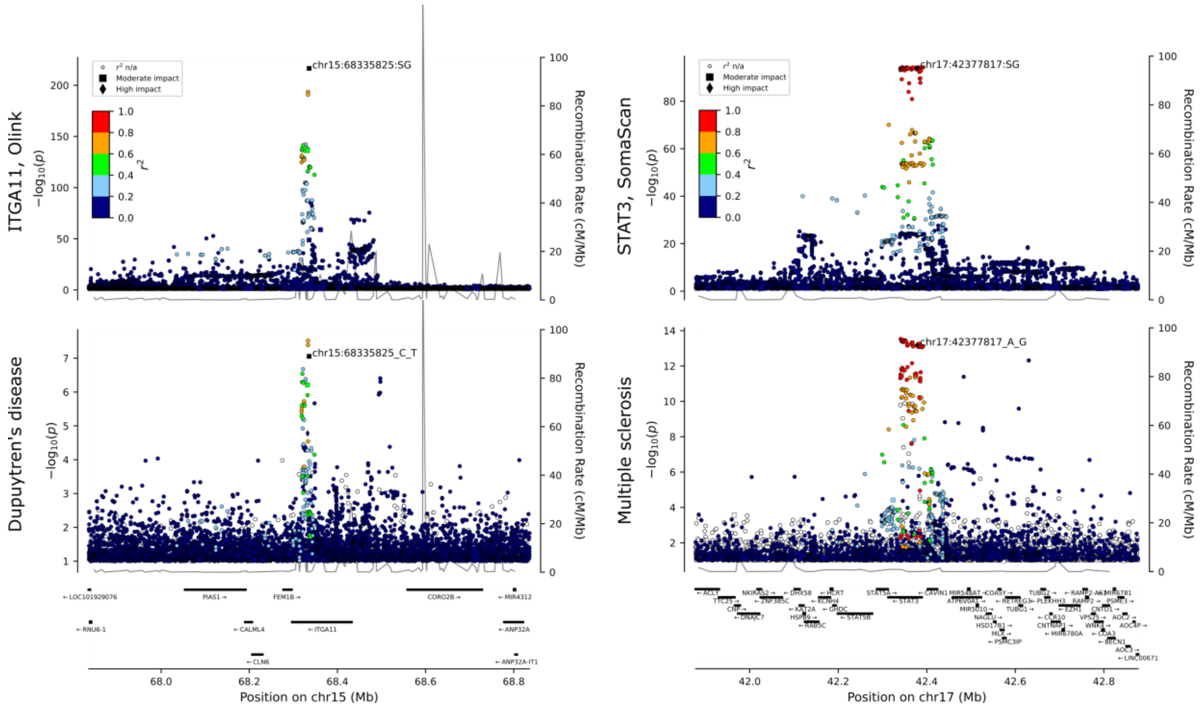

Supplementary Figure SF8: Left: Association at *ITGA11* locus between variants and ITGA11 levels measured using Olink Explore, and Dupuytren´s disease. All r^2 are shown to the same variant.

Right: Association at *STAT3* locus between variants and STAT3 levels measured using SomaScan v4 (top) and multiple sclerosis risk (bottom). All r^2 are shown to the same variant.

p-values were based on a two-sided likelihood ratio test and not adjusted for multiple comparisons.

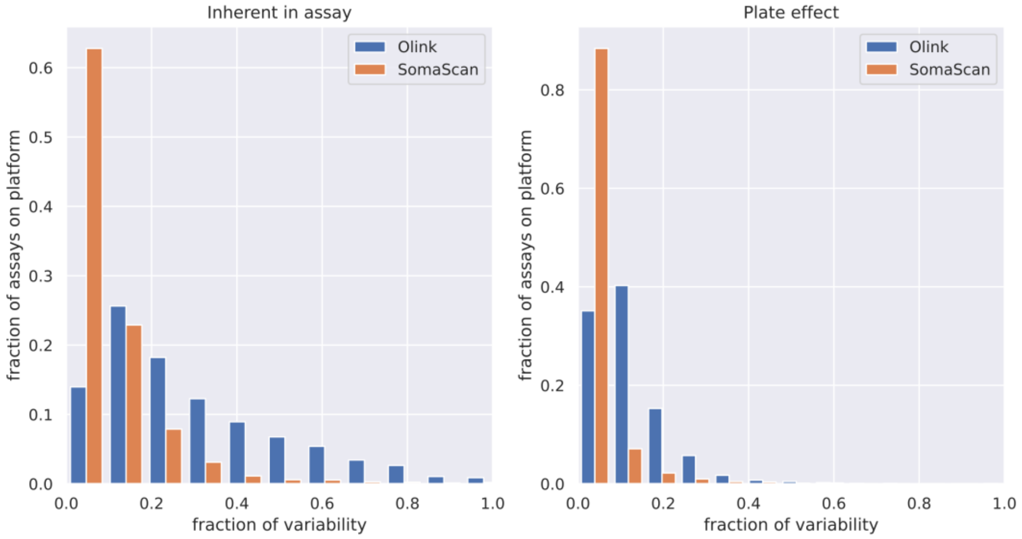

Supplementary Figure SF9: Fraction of total variance of the assay inherent in the assay itself (left) and due to plate effect (right). Estimated using control samples from both platforms.

References

1. Katz, D. H. *et al.* Proteomic profiling platforms head to head: Leveraging genetics and clinical traits to compare aptamer- and antibody-based methods. *Sci. Adv.* **8**, eabm5164 (2022).
2. Raffield, L. M. *et al.* Comparison of Proteomic Assessment Methods in Multiple Cohort Studies. *Proteomics* **20**, e1900278 (2020).
3. Candia, J. *et al.* Assessment of Variability in the SOMAscan Assay. *Sci. Rep.* **7**, 1–13 (2017).
4. Ferkingstad, E. *et al.* Large-scale integration of the plasma proteome with genetics and disease. *Nat. Genet.* **53**, 1712–1721 (2021).
5. Jougasaki, M., Wei, C. M., McKinley, L. J. & Burnett, J. C. Elevation of circulating and ventricular adrenomedullin in human congestive heart failure. *Circulation* **92**, 286–289 (1995).
6. Folkersen, L. *et al.* Genomic and drug target evaluation of 90 cardiovascular proteins in 30,931 individuals. *Nat. Metab.* **2**, 1135–1148 (2020).
7. Sun, B. B. *et al.* Genomic atlas of the human plasma proteome. *Nature* **558**, 73–79 (2018).
8. Pietzner, M. *et al.* Synergistic insights into human health from aptamer- and antibody-based proteomic profiling. *Nat. Commun. 2021 121* **12**, 1–13 (2021).
9. Chen, M.-H. *et al.* Trans-ethnic and Ancestry-Specific Blood-Cell Genetics in 746,667 Individuals from 5 Global Populations. *Cell* **182**, 1198-1213.e14 (2020).
10. Johnson, A. D. *et al.* Genome-wide meta-analyses identifies seven loci associated with platelet aggregation in response to agonists. *Nat. Genet.* **42**, 608–613 (2010).
11. Pietzner, M. *et al.* Genetic architecture of host proteins involved in SARS-CoV-2 infection. *Nat. Commun.* **11**, 6397 (2020).
12. Elliott, P., Peakman, T. C., & on behalf of UK Biobank. The UK Biobank sample handling and storage protocol for the collection, processing and archiving of human blood and urine. *Int. J. Epidemiol.* **37**, 234–244 (2008).
13. Mussbacher, M. *et al.* Optimized plasma preparation is essential to monitor platelet-stored molecules in humans. *PLoS ONE* **12**, e0188921 (2017).

14. Ferkingstad, E. *et al.* Genome-wide association meta-analysis yields 20 loci associated with gallstone disease. *Nat. Commun.* **9**, 5101 (2018).

15. Pietzner, M. *et al.* Mapping the proteo-genomic convergence of human diseases. *Science* **374**, eabj1541 (2021).

16. de Rojas, I. *et al.* Common variants in Alzheimer's disease and risk stratification by polygenic risk scores. *Nat. Commun.* **12**, 3417 (2021).

17. Buniello, A. *et al.* The NHGRI-EBI GWAS Catalog of published genome-wide association studies, targeted arrays and summary statistics 2019. *Nucleic Acids Res.* **47**, D1005–D1012 (2019).

18. International Multiple Sclerosis Genetics Consortium. Multiple sclerosis genomic map implicates peripheral immune cells and microglia in susceptibility. *Science* **365**, eaav7188 (2019).

19. Zhang, Y., Liu, Q., Yang, S. & Liao, Q. CD58 Immunobiology at a Glance. *Front. Immunol.* **12**, 705260 (2021).

20. Ng, M. *et al.* A Genome-wide Association Study of Dupuytren Disease Reveals 17 Additional Variants Implicated in Fibrosis. *Am. J. Hum. Genet.* **101**, 417–427 (2017).

21. Sakaue, S. *et al.* A cross-population atlas of genetic associations for 220 human phenotypes. *Nat. Genet.* **53**, 1415–1424 (2021).

Index of supplementary tables

ST2: Assays on the Olink platform

oid: Internal Olink ID of assay
uniprot: UniProt ID of the protein targeted by the assay
target_full_name: Name of the protein targeted by the assay according to Olink
gene_name: Name of the gene encoding the protein targeted by the assay
dilution: Dilution group of the assay (1:n)
cv: CV of repeated measurements of the same sample
rand_cv: CV of randomly chosen pairs of samples from the assay
rand_cv_iceland_1k: rand_cv on the Icelandic 1K data set

ST3: Assays on the SomaScan platform

seqid: Internal SomaScan ID of the assay
uniprot: UniProt ID of the protein targeted by the assay
target_name: Name of the protein targeted by the assay according to SomaLogic
target_full_name: Full name of the protein targeted by the assay according to SomaLogic
gene_name: Name of the gene encoding the protein targeted by the assay
dilution: Dilution group of the assay (1:n)
nonnorm_cv: CV of repeated measurements of the same sample, non-SMP normalized
nonnorm_rand_cv: CV of randomly chosen pairs of samples from the assay, non-SMP normalized
norm_cv: CV of repeated measurements of the same sample, SMP normalized
norm_rand_cv: CV of randomly chosen pairs of samples from the assay, SMP normalized
rand_cv_iceland_1k: norm_rand_cv on the Icelandic 1K data set, SMP normalized
on_1.2k: Was the protein included on the SomaScan 1.2k platform?

ST6: Correlation between assays on the Olink and SomaScan platforms

seqid: Internal SomaScan ID of the SomaScan assay
oid: Internal Olink ID of the Olink assay
uniprot: UniProt ID of the target protein
target: Name of the target protein
target_name: Full name of the target protein
gene_name: Name of the gene encoding the protein targeted by the assays
olink_nonnorm_corr: Spearman correlation between protein levels measured with Olink and non-SMP normalized SomaScan assay
olink_nonnorm_pval: Significance of olink_nonnorm_corr
olink_smpnorm_corr: Spearman correlation between protein levels measured with Olink and SMP normalized SomaScan assay
olink_smpnorm_pval: Significance of olink_smpnorm_corr
nonnorm_smpnorm_corr: Spearman correlation between protein levels measured with SMP normalized and non-SMP normalized SomaScan assay
nonnorm_smpnorm_pval: Significance of nonnorm_smpnorm_corr
olink_panelset: Inclusion of assay on Olink Explore 1536 (1) or Olink Expansion (2)

smp_quantile_in_olink: Quantile of correlation of the corresponding assay among all SomaScan assays to the relevant Olink assay (SMP normalized)
smp_quantile_in_somascan: Quantile of correlation of the corresponding assay among all Olink assays to the relevant SomaScan assay (SMP normalized)
nonnorm_quantil_in_olink: Quantile of correlation of the corresponding assay among all SomaScan assays to the relvant Olink assay (non-SMP normalized)
nonnorm_quantile_in_somascan: Quantile of correlation of the corresponding assay among all Olink assays to the relevant SomaScan assay (non-SMP normalized)

ST11: Median correlation of assays by tissue of preferential expression

tissue: Tissue of preferential expression (according to Human Protein Atlas)
median_correlation: Median of correlation coefficients for proteins preferentially expressed inthe tissue
mean_correlation: Mean of correlation coefficients for proteins preferentially expressed in the tissue
n_assay_pairs: Number of assay pairs targeting proteins preferentially expressed in the tissue
n_proteins: Number of unique proteins targeted by both platforms and preferentially expressed in the tissue

ST13: Correlations of protein levels measured on Olink with subject age, sex, and sample age in the UKB data sets

oid: Internal Olink ID of the assay
uniprot: UniProt ID of the targeted protein
gene_name: Name of the gene encoding the protein
beta_age_bi: Beta of subject age in the British-Irish ancestry group
pval_age_bi: Significance of beta_age_bi
beta_age_af: Beta of subject age in the African ancestry group
pval_age_af: Significance of beta_age_af
beta_age_sa: Beta of subject age in the South-Asian ancestry group
pval_age_sa: Significance of beta_age_sa
beta_sex_bi: Beta of subject sex in the British-Irish ancestry group
pval_sex_bi: Significance of beta_sex_bi
beta_sex_af: Beta of subject sex in the African ancestry group
pval_sex_af: Significance of beta_sex_af
beta_sex_sa: Beta of subject sex in the South-Asian ancestry group
pval_sex_sa: Significance of beta_sex_sa
beta_sample_age: Beta of sample age, all ancestry groups
pval_sample_age: Significance of beta_sample_age

ST14: Correlations of protein levels measured on SomaScan with subject age, sex, and sample age in the Iceland data set

seqid: Internal SomaScan ID of the assay
gene_name: Name of the gene encoding the protein targeted by the assay

beta age no normalization: Beta of subject age (non-SMP normalized)
beta age SMP normalization: Beta of subject age (SMP normalized)
pval age no normalization: Significance of beta age no normalization
pval age SMP normalization: Significance of beta age SMP normalization
beta sex no normalization: Beta of subject sex (non-SMP normalized)
beta sex SMP normalization: Beta of subject sex (SMP normalized)
pval sex no normalization: Significance of beta sex no normalization
pval sex SMP normalization: Significance of beta sex SMP normalization
beta sample age no normalization: Beta of sample age (non-SMP normalized)
pval sample age no normalization: Significance of beta sample age no normalization
beta sample age no normalization adjusted for site: Beta of sample age (non-SMP normalized), adjusted for sample site (UVS or HERA)
pval sample age no normalization adjusted for site: Significance of beta sample age no normalization adjusted for site

ST15: Significant associations between protein levels on Olink and phenotypes in the UKB data set

oid: Internal Olink ID for the assay
uniprot: The UniProt ID of the protein targeted by the assay
gene_name: The name of the gene encoding the protein targeted by the assay
icd10: ICD10 code of the phenotype
phenotype: Name of the phenotype
OR_BI: Odds ratio in the BI ancestry group
pval_BI: Significance of OR_BI
nbi: Number of affected individuals in the BI ancestry group
OR_AF: Odds ratio in the AF ancestry group
pval_AF: Significance of OR_AF
naf: Number of affected individuals in the AF ancestry group
OR_SA: Odds ratio in the SA ancestry group
pval_SA: Significance of OR_SA
nsa: Number of affected individuals in the SA ancestry group
beta_BI: Beta of the association in the BI ancestry group
beta_AF: Beta of the association in the AF ancestry group
beta_SA: Beta of the association in the SA ancestry group

ST16: Significant associations between protein levels on SomaScan and phenotypes in the Iceland data set

seqid: Internal SomaLogic ID of the assay
gene_name: Name of the gene encoding the protein targeted by the assay
beta no normalization: Beta of the association (non-SMP normalized)
pval no normalization: Significance of beta no normalization
beta SMP normalization: Beta of the association (SMP normalized)
pval SMP normalization: Significance of beta SMP normalization
Phenotype: Name of the phenotype

ListType: Quantitative or case-control phenotype
ncases: Number of cases (case-control phenotypes)
ncontrols: Number of controls (case-control phenotypes)
nmeasured: Number of measurements (quantitative phenotypes)

ST17: Significant associations of protein levels to IBD, both Olink (UKB-BI) and SomaScan (Iceland)

oid: Internal Olink assay ID
OR_olink: Odds ratio for the association on the Olink platform
pval_olink: Significance of the association on the Olink platform
rank_olink: Rank of the association on the Olink platform
seqid: Internal SomaLogic assay ID
uniprot: UniProt ID of the target protein
Gene_name: The name of the gene encoding the target protein
TargetFullName: Full name of the targeted protein
OR_somascan: Odds ratio for the association on the SomaScan platform
pval_somascan: Significance of the association on the SomaScan platform
rank_somascan: Rank of the association on the Olink platform
correlation: Correlation between protein levels measured using the assays
targeted_by: Indicator of which platforms target the protein (olink_only, somascan_only or olink_somascan)
cis_pqtl_olink: Indicator if the protein has a *cis* pQTL on the Olink platform
cis_pqtl_somascan: Indicator if the protein has a *cis* pQTL on the SomaScan platform

ST18: pQTLs detected on the SomaScan platform in the Iceland data set using SMP normalized data

locusID global: Global identifier of the pQTL (set of variants with $r^2 \geq 0.80$)
locusID prot: Protein-specific indicator of sentinel pQTL association
gene prot: Name of the gene encoding the protein
chr prot: Chromosome of the gene encoding the protein
strand prot: Strand of the gene encoding the protein
TSS prot: Transcription start site of the gene encoding the protein
shortname prot: Name of the targeted protein
longname prot: Full name of the targeted protein
UniProt: UniProt ID of the targeted protein
variant: RS name of the variant associating with the protein
LD class size: Number of variants in high LD (r2 > 0.8) with the variant
LD class: Variants in high LD (r2 > 0.8) with the variant
chr var: Chromosome of the variant
pos var: Position of the variant
region start: Start of the region containing the sentinel variant
region end: End of the region containing the sentinel variant
Amin: Minor allele of the variant
Amaj: Major allele of the variant

Info: Imputation info of the variant
MAF PC: Minor allele frequency (%)
cis trans: Is the pQTL cis or trans for the protein
distTSS: Distance between variant and transcription start site
closest genes: List of genes close to the variant
cis eqtl coding genes: List of genes that harbor a protein-altering (coding) or eQTL variant in high LD with pQTL variant
beta adj: Effect (in standard deviations; SD) on protein levels; adjusted for variants of higher rank in the step-wise conditional analysis (if any)
beta unadj: Marginal/unadjusted effect (in SD) on protein levels
mLog10pval adj: Negative base-10 logarithm corresponding to beta adj, e.g. a value of 12 means that the p-value is 10^{-12}
mLog10pval unadj: Negative base-10 logarithm corresponding to beta unadj, e.g. a value of 12 means that the p-value is 10^{-12}
rank locus: Rank in step-wise conditional analysis, where sentinel variants have rank=1 and secondary variants rank 2, 3, and so on
count locus: Number of significant variants in step-wise conditional analysis
count cis eQLT same gene: For cis pQTL associations, the number of cis-eQTL in high LD for the gene encoding the protein with affected leves (NA for trans pQTL associations)
prop same direction cis eqtl same gene: For cis pQTL associations, the proportion of cis-eQTL in high LD that have the same direction of effect for the pQTL and the eQTL for the gene encoding the protein with affected levels (NA for trans pQTL associations);
count cis eQTL different gene: For cis pQTL associations, the number of cis-eQTL in high LD for genes other than the gene encoding the protein with affected leves (NA for trans pQTL associations).
any coding same gene: For cis-pQTL associations, «Y» if there are protein altering variants (PAV) in high LD with the pQTL variant for the gene encoding the affected protein, «N» otherwise (NA for trans pQTL associations).
any coding diff gene: For cis-pQTL associations, «Y» if there are PAV in high LD with the pQTL variant for other genes than the gene encoding the affected protein, «N» otherwise (NA for trans pQTL associations).
Rank Proteins per pQTL: Ranks (in terms of significance of the sentinel pQTL association) of the associated proteins per pQTL.
Count Proteins per pQTL: Count of the associated proteins per pQTL
Rank pQTL per protein: Rank of the pQTL among all pQTL associated with protein
Count pQTL per protein: Number of pQTLs associated with protein
Reported: «Y» if pQTL association is previously reported (for same protein and any variant in high LD), «N» otherwise.
Reported rsid: RSid of the variant reported (if any)
Reported p: P-value of the reported association
Reported PMID: PMID of the reporting article
Reported r2: r2 between the reported variant and the pQTL
SeqId: Internal SomaLogic ID of the assay
r2 joint pQTL: Variance explained (r2) by the effect in a joint model for all variants at the locus

Effect in joint model: Effect estimate in a joint model for all variants at the locus
P-value joint model: Significance of the effect for the joint model

ST19: pQTLs detected on the SomaScan platform in the Iceland data using non-normalized data

See S10

ST20: pQTLs detected on the Olink platform in the UKB-BI data set

Chrom: Chromosome of the variant
Pos: Position of the variant
OlinkID: Internal Olink ID of the assay
Affected Protein Gene Name: Name of the gene encoding the associated protein
pQTL ID: Global identifier of the pQTL
Region ID prot: Protein-specific indicator of sentinel pQTL association.
CisOrTrans: Indicator if the pQTL association is cis or trans
marker: The variant associating with the protein levels
rsName: RS name of the variant
MAF PC: Minor allele frequency of the variant (%)
Info: Imputation info of the variant
Amin: Minor allele of the variant
Amaj: Major allele of the variant
Log10 pval gc cor unadj: Significance of the association without adjusting for other signals at the locus. Negative log10 of the p-value.
Log10 pval gc cor adj: Significance of the association, adjusted for higher-rank signals at the locus. Negative log10 of the p-value.
Effect Amin unadj: Effect of the minor allele of the variant without adjusting for other signals at the locus.
Effect Amin adj: Effect of the minor allele of the variant, adjusted for higher-rank signals at the locus.
Rank Within Locus: Rank in step-wise conditional analysis, where sentinel variants have rank=1 and secondary variants rank 2, 3, and so on.
Number Within Locus: Number of significant variants in step-wise conditional analysis.
Sentinel Marker: The variant at the locus with the most significant association with the protein
Affected Protein Gene chrom: Chromosome of the gene encoding the protein
Affected Protein Gene chromstart: Start of the gene
Affected Protein Gene chromend: End of the gene
strand: Strand of the gene
TopAtLocus: Indicator if the pQTL is the sentinel signal at the locus
Affected Protein Gene TSS: Transcription start site for the gene
SameChrom: Indicator if the gene and variant are on the same chromosome
distanceTSSvariant: Distance between transcription start site and variant
rank proteins per pQTL: rank (in terms of significance of the sentinel pQTL association) of the associated proteins per pQTL.
count proteins per pQTL: Count of the associated proteins per pQTL.

rank pQTL per protein: Rank of the pQTL among all pQTL associated with protein.
count pQTL per protein: Number of pQTL associated with protein.
r2 sentinel: r2 between the variant and the sentinel variant at the locus
cis eQTL genes: Genes that harbor an eQTL variant in high LD with pQTL variant
PAV genes: Genes that harbor a protein-altering (coding) variant in high LD with pQTL variant
UniProt: UniProt ID of the protein
cis eQTL same gene: For cis-pQTL associations, «Y» if there are cis-eQTL in high LD with the pQTL variant for other genes than the gene encoding the affected protein, «N» otherwise (NA for trans pQTL associations)
PAV same gene: For cis-pQTL associations, «Y» if there are protein altering variants (PAV) in high LD with the pQTL variant for other genes than the gene encoding the affected protein, «N» otherwise (NA for trans pQTL associations),
Olink version: Version of the Olink platform (Explore or Expansion)
protein on soma: Indicator if the protein is also targeted by the SomaScan platform
r2 Joint PQTL: Variance explained (r2) by the effect in a joint model for all variants at the locus
Coef joint: Effect of the model in a joint model of all independent variants at the locus
Pval joint: Significance of the variant in a joint model of all independent variants at the locus

ST21: pQTLs detected on the Olink platform in the UKB-AF data set

Chrom: Chromosome of the variant
Pos: Position of the variant
marker: The variant associating with the protein levels
OlinkID: The internal Olink ID of the assay
mlog10pval: Significance of the association between the variant and the protein levels. Negative log10 of the p-value.
Effect: The effect of the variant on the protein levels.
rsName: The RS name of the variant
MAF_PC: Minor allele frequency of the variant (%)
Info: Imputation info of the variant
Amin: Minor allele of the variant
Amaj: Major allele of the variant
close_genes: Genes close to the variant
prot_gene: The gene encoding the protein targeted by the assay
prot_Chrom: Chromosome of the gene encoding the protein targeted by the assay
prot_TSS: Transcription start site of the gene encoding the protein targeted by the assay
cis_trans: Indicator if the pQTL is cis or trans for the protein
Olink_set: Indicator to which set of Olink panels the assay belongs
any_PAV_same_gene: For cis-pQTL associations, «Y» if there are protein altering variants (PAV) in high LD with the pQTL variant for other genes than the gene encoding the affected protein, «N» otherwise (NA for trans pQTL associations)

ST22: pQTLs detected on the Olink platform in the UKB-SA data set

See S13

ST23: Percentage of measurements below the LOD from each assay on the Olink platform the UKB data

oid: Internal Olink ID of assay
perc.below.lod: Percentage of values of assay below limit of detection

ST24: Comparison between pQTLs detected on Olink in the UKB-AF and UKB-BI data sets

Chrom: Chromosome of the variant
Pos_AFR: Position of the variant in the UKB-AF data set
Pos_EUR: Position of the variant in the UKB-BI data set
marker_AFR: Name of the variant in the UKB-AF data set
OlinkID: Internal Olink ID of the assay
mlog10pval_AFR: Significance of the association in the UKB-AF data set. Negative log10 of p-value.
Effect_AFR: Effect of the association in the UKB-AF data set.
rsName_ARF: RS name of the variant in the UKB-AF data set
MAF_PC_AFR_XAF: Minor allele frequency of the variant in the UKB-AF data set
Info_AFR: Imputation info in the UKB-AF data set
Amin_AFR: Minor allele in the UKB-AF data set
Amaj_AFR: Major allele in the UKB-AF data set
close_genes_AFR: Genes that are close to the variant in the UKB-AF data set
prot_gene: Name of the gene encoding the targeted protein
prot_Chrom: Chromosome of the gene encoding the targeted protein
prot_TSS: Transcription start site of the gene encoding the targeted protein
cis_trans: Indicator if the pQTL is cis or trans for the protein
Olink_set: Indicator if the assay is in the 1536 or Expansion set of assays
MAF_flip: Indicator if the minor alleles in the UKB-AF and UKB-BI data sets are different
MAF_PC_AFR_XBI: Minor allele frequency of the pQTL discovered in the UKB-AF data set in the UKB-BI imputation
MAF_AFR_div_XAF_XBI: Ratio MAF_PC_AFR_XAF/MAF_PC_AFR_XBI
distance: Distance between pQTL detected in UKB-AF data-set and pQTL detected in UKB-BI data set
marker_EUR: Name of the variant in the UKB-BI data set
mlog10pval_EUR: Significance of the association in the UKB-BI data set. Negative log10 of p-value.
Effect_EUR: Effect of the association in the UKB-BI data set
rsName_EUR: RS name of the marker in the UKB-BI data set
MAF_PC_EUR: Minor allele frequency of the marker in the UKB-BI data set
Info_EUR: Imputation info in the UKB-BI data set
Amin_EUR: Minor allele in the UKB-BI data set
Amaj_EUR: Minor allele in the UKB-BI data set
r2_XAF: r2 between the variant in the UKB-AF data set and the UKB-BI data set, calculated in the UKB-AF data set
r2_XBI: r2 between the variant in the UKB-AF data set and the UKB-BI data set, calculated in the UKB-BI data set

ldclass_AFR_marker: The LD class of the variant in the UKB-AF data set
ldclass_AFR_size: Size of the LD class of the variant in the UKB-AF data set
ldclass_AFR_rsname: RS names of the variants in the LD class in the UKB-AF data set
ldclass_EUR_marker: The LD class of the variant in the UKB-BI data set
ldclass_EUR_size: Size of the LD class of the variant in the UKB-BI data set
ldclass_EUR_rsname: RS names of the variants in the LD class in the UKB-BI data set
size_ldclass_overlap: Number of variants common to the UKB-AF and UKB-BI LD classes
rsname_ldclass_overlap: RS names of the variants common to the UKB-AF and UKB-BI LD classes

ST25: Comparison between pQTLs detected on Olink in the UKB-SA and UKB-BI data sets

See S17

ST26: Proportion of proteins with cis pQTLs depending on dilution, subcellular location and overlap between platforms

Platform: Proteomics platform (SomaScan or Olink)
Value Type: Protein dilution, subcellular location or overlap
Value: Value to classify by
Proportion of proteins with cis-pQTL: Proportion of proteins with cis pQTL
Number of proteins in group: Total number of proteins in the group

ST27: Comparison of results for each protein targeted by at least one of the platforms

UniProt: UniProt ID of the target protein
OlinkID: Internal Olink ID of the targeted protein
SeqId: Internal SomaLogic ID of the targeted protein
Gene: Name of the gene encoding the targeted protein
olink smp corr: Spearman correlation between the Olink and SomaScan assays (SMP normalization)
olink smp pval: Significance of olink smp corr
Targeted Olink: Indicator if the protein is targeted by the Olink Explore 3072 platform
Targeted Soma: Indicator if the protein is targeted by the SomaScan v4 platform
cis pQTL olink: Indicator if the protein has a cis pQTL in the UKB-BI Olink data
PAV olink: Indicator if the protein has a pQTL in high LD with a PAV in the UKB-BI Olink data
eQTL olink: Indicator if the protein has a pQTL in high LD with a cis eQTL in the UKB-BI Olink data
Dilution olink: Dilution group on the Olink platform
cis pQTL soma: Indicator if the protein has a cis pQTL in the Icelandic SomaScan data
PAV soma: Indicator if the protein has a pQTL in high LD with a PAV in the Icelandic SomaScan data
eQTL soma: Indicator if the protein has a pQTL in high LD with a cis eQTL in the Icelandic SomaScan data
Dilution soma: Dilution group on the SomaScan platform
location: Subcellular location of the protein
RNA tissue specificity: RNA tissue specificity of the protein
RNA tissue specific nTPM: RNA tissue specific normalized transcript expression values
Olink set: Olink assay set (1536 or Expansion)

N platforms tested: Number of platforms (SomaScan or Olink) targeting the protein
N platforms with cis pQTL: Number of platforms on which a cis pQTL was detected for the protein
cis pQTL on both and high correlation (>0.5): Indicator if the protein has a cis pQTL on both platforms and the correlation between levels is high
r2 top cis pQTL: r2 between the top cis pQTLs on each platform
confidence tier: Indicates the level of confidence. Tier 1: cis pQTL on both platforms, high correlation of levels. Tier 2: cis pQTL on at least one platform. Tier 3: cis pQTL on neither platform.

ST28: Correspondence of pQTLs between the Olink (UKB-BI) and SomaScan (Iceland) platforms

Chrom: Chromosome of the Olink and SomaScan pQTLs
OLINK_Pos: Position of the Olink pQTL
r2_UK: r^2 between Olink and SomaScan pQTL, based on UKB imputation
dist_UK_IS: Distance between Olink and SomaScan pQTL
OLINK_OlinkID: Internal Olink assay ID
OLINK_UniProt: UniProt ID of protein
OLINK_gene_prot: Gene symbol of gene encoding protein
OLINK_pQTL_ID: Global identifier of the Olink pQTL
OLINK_cis_trans: Indicator if the pQTL association is cis or trans
OLINK_rsName: RS name of the Olink pQTL
OLINK_MAF_PC: Minor allele frequency of the Olink pQTL (%)
OLINK_close_genes: Closest genes to the Olink pQTL
OLINK_mLog10pval_unadj: Significance of the Olink pQTL association without adjusting for other signals at the locus. Negative log10 of the p-value.
OLINK_mLog10pval_adj: Significance of the Olink pQTL association, adjusted for higher-rank signals at the locus. Negative log10 of the p-value.
OLINK_beta_unadj: Effect of the minor allele of the Olink pQTL variant without adjusting for other signals at the locus.
OLINK_beta_adj: Effect of the minor allele of the Olink pQTL variant, adjusted for higher-rank signals at the locus.
OLINK_rank_locus: Rank in step-wise conditional analysis for Olink pQTL, where sentinel variants have rank=1 and secondary variants rank 2, 3, and so on
OLINK_count_locus: Number of significant variants in step-wise conditional analysis for Olink pQTL
OLINK_rank_proteins_per_pQTL: For Olink pQTL: Rank (in terms of significance of the sentinel pQTL association) of the associated proteins per pQTL.
OLINK_count_proteins_per_pQTL: For Olink pQTL: Count of the associated proteins per pQTL
OLINK_rank_pQTL_per_protein: For Olink pQTL: Rank of the pQTL among all pQTL associated with protein.
OLINK_count_pQTL_per_protein: For Olink pQTL: Number of pQTL associated with protein
SOMA_Pos: Position of the SomaScan pQTL
SOMA_locusID_global: Global identifier of the SomaScan pQTL
SOMA_SeqId: Internal SomaScan assay ID
SOMA_variant: RS name of the SomaScan pQTL
SOMA_MAF_PC: Minor allele frequency of the SomaScan pQTL (%)

SOMA_cis_trans: Indicator if the pQTL association is cis or trans
SOMA_closest_genes: Closest genes to the Olink pQTL
SOMA_cis_eqtl_coding_genes: List of genes that harbor a protein-altering (coding) or eQTL variant in high LD with pQTL variant
SOMA_beta_unadj: Marginal/unadjusted effect (in SD) of SomaScan pQTL on protein levels
SOMA_beta_adj: Effect (in standard deviations; SD) of SomaScan pQTL on protein levels; adjusted for variants of higher rank in the step-wise conditional analysis (if any)
SOMA_mLog10pval_unadj: Negative base-10 logarithm corresponding to beta unadj, e.g. a value of 12 means that the p-value is 10^{-12}
SOMA_mLog10pval_adj: Negative base-10 logarithm corresponding to beta adj, e.g. a value of 12 means that the p-value is 10^{-12}
SOMA_rank_locus: Rank in step-wise conditional analysis for SomaScan pQTL, where sentinel variants have rank=1 and secondary variants rank 2, 3, and so on
SOMA_count_locus: Number of significant variants in step-wise conditional analysis for Olink pQTL
SOMA_rank_proteins_per_pQTL: For SomaScan pQTL: Rank (in terms of significance of the sentinel pQTL association) of the associated proteins per pQTL.
SOMA_count_proteins_per_pQTL: For SomaScan pQTL: Count of the associated proteins per pQTL
SOMA_rank_pQTL_per_protein: For SomaScan pQTL: Rank of the pQTL among all pQTL associated with protein.
SOMA_count_pQTL_per_protein: For SomaScan pQTL: Number of pQTL associated with protein

ST29: Pleiotropic trans pQTLs on the Olink (UKB-BI) and SomaScan (Iceland) platforms

Chrom: Chromosome of variant
Olink Pos (hg38): Position of associated variant on Olink
Highly Pleiotropic Olink: Indicator if the variant is highly pleiotropic on Olink
Highly Pleiotropic Soma SMP: Indicator if the variant is highly pleiotropic on SomaScan with SMP normalization
Highly Pleiotropic Soma PC0: Indicator if the variant is highly pleiotropic on SomaScan without SMP normalization
r2 SMP Olink: r2 between the corresponding variants in the Olink data and the SMP normalized SomaScan data
distance SMP olink: Distance between the corresponding variants in the Olink data and the SMP normalized SomaScan data
r2 PC0 Olink: r2 between the corresponding variants in the Olink data and the non-SMP normalized SomaScan data
distance PC0 olink: Distance between the corresponding variants in the Olink data and the non-SMP normalized SomaScan data
Olink nprot: Number of proteins associating with the variant in the Olink data
Soma SMP nprot: Number of proteins associating with the variant in the SMP normalized SomaScan data
Soma PC0 nprot: Number of proteins associating with the variant in the non-SMP normalized SomaScan data
nprot SMP div nprot Olink: Soma SNP nprot / Olink nprot
nprot PC0 div nprot Olink: Soma PC0 nprot / Olink nprot

nprot SMP div nprot PC0: Soma SMP nprot / Soma PC0 nprot
Olink Name: Name of associated variant in the Olink data
Olink rsName: RS name of the associated variant in the Olink data
Olink MAF PC: Minor allele frequency of the associated variant in the Olink data (%)
Olink close genes: Genes close to the associated variant in the Olink data
Soma SMP Pos: Position of the associated variant in the SomaScan data using SMP normalization
Soma SMP Name: Name of the associated variant in the SomaScan data using SMP normalization
Soma SMP rsName: RS name of the associated variant in the SomaScan data using SMP normalization
Soma SMP MAF PC: Minor allele frequency of the associated variant in the SomaScan data using SMP normalization (%)
Soma SMP close genes: Genes close to the associated variant in the SomaScan data using SMP normalization
Soma PC0 Pos: Position of the associated variant in the non-SMP normalized SomaScan data
Soma PC0 Name: Name of the associated variant in the non-SMP normalized SomaScan data
Soma PC0 rsName: RS name of the associated variant in the non-SMP normalized SomaScan data
Soma PC0 MAF PC: Minor allele frequency of the associated variant in the non-SMP normalized SomaScan data (%)
Soma PC0 close genes: Genes close to the associated variant in the non-SMP normalized SomaScan data

ST30: Correspondence between pleiotropic trans pQTLs on the Olink (UKB-BI) and SomaScan (Iceland) platforms

Chrom: Chromosome of the variant
Pos (hg38): Position of the variant
rsName: RS name of the variant
MAF (%): Minor allele frequency of the variant (%)

ST31: Cis pQTLs in high LD with disease-associated variants from the GWAS catalog

Chrom: Chromosome of the variant
Pos: Position of the variant
variant: Variant name
pQTL ID: ID of the pQTL (including platform)
Olink ID: Internal Olink ID of the assay
*SeqId: I*nternal SomaLogic ID of the assay
gene prot: Name of the gene encoding the targeted protein
close genes: Genes close to the variant
source: Source of the pQTL (Olink data, SMP normalized SomaScan data, non-SMP normalized SomaScan data)
r2: r2 between the pQTL variant and the variant in the GWAS catalog
GWAS EFO: Experimental Factor Ontology reported in the GWAS catalog
GWAS RSID: RS name of the variant in the GWAS catalog
GWAS P: Significance of the association reported in the GWAS catalog

GWAS PMID: PubMed ID of the article reporting the association in the GWAS catalog
GWAS PHENO: Phenotype reported in the GWAS catalog
GWAS PHENO TEXT: Information describing context of GWAS phenotype (e.g. females, smokers).
pQTL log10 p: Significance of the pQTL association. Negative log10 of p-value.
pQTL Effect amin: Effect of the minor allele of the pQTL
MAF PC: Minor allele frequency of the pQTL variant (%)
credible set: Indicator if the GWAS catalog variant is included in the credible set of variants for the pQTL

ST32: Trans pQTLs in high LD with disease-associated variants from the GWAS catalog

Chrom: Chromosome of the variant
pQTL ID: ID of the pQTL (including platform)
Olink ID: Internal ID of the Olink assay
gene prot: Name of the gene encoding the protein
N prot: Number of proteins associating with the variant
GWAS EFO: Experimental Factor Ontology reported in the GWAS catalog
N GWAS EFO: Number of EFOs reported in the GWAS catalog
GWAS RSID: RS name of variants reported in the GWAS catalog
MAF PC: Minor allele frequency of the pQTL variant (%)
prop in credible set: Proportion of the GWAS catalog variants included in the credible set of variants for the pQTL
source: Platform where the pQTL was detected
gene at locus: Closest gene to pQTL variant
linked protein(s): Proteins among the associated proteins ("gene prot") that are linked to "gene at locus" according to STRING database (see below)
STRING score(s): STRING scores corresponding to linked proteins

ST33: Summary of the numbers of pQTLs associating with disease-associated variants from the GWAS catalog using LD and / or credible sets

Platform: Platform where the pQTL is detected
Entity: Objects being counted
Total count r2 only: Number of pairs of items where the associated variants are in high LD
Cis only count r2 only: Number of pairs of items involving only cis pQTL associations where the associated variants are in high LD
Trans only count r2 only: Number of pairs items involving only trans pQTL associations where the associated variants are in high LD
Cis and Trans count r2 only: Number of pairs items involving both cis and trans pQTL associations where the associated variants are in high LD
Total count r2 and credible set: Number of pairs of items where the associated variants are in high LD and the GWAS catalog variant is in the credible set for the pQTL association.
Cis only count r2 and credible set: Number of pairs of items involving only cis pQTL associations where the associated variants are both in high LD and the GWAS catalog variant is in the credible set for the pQTL association.

Trans only count r2 and credible set: Number of pairs of items involving only cis pQTL associations where the associated variants are both in high LD and the GWAS catalog variant is in the credible set for the pQTL association.

Cis and Trans count r2 and credible set: Number of pairs items involving both cis and trans pQTL assocations where the associated variants are in high LD and the GWAS catalog variant is in the credible set for the pQTL association.

ST35: Replication of pQTLs detected in the Olink data (UKB-BI) in the Icelandic Olink data (n=1,514)

Chrom: Chromosome of variant
Pos: Position of variant
Name: Name of variant
OlinkID: Internal Olink ID of assay
Affected_Protein_Gene_name: Name of the gene encoding the protein
pQTL_ID: ID of the pQTL
cis_trans: Indicator if the pQTL is cis or trans for the protein
rsName: RS name of the variant
MAF_PC: Minor allele frequency of the variant (%)
Info: Imputation info of the variant
Amin: Minor allele of the variant
Amaj: Major allele of the variant
close_genes: Genes close to the variant
Log10_Pval_UK: Significance of the pQTL in the UKB-BI data. Negative log10 of the p-value.
Effect_Amin_UK: Effect of the minor allele in the UKB-BI data.
Pval_Iceland: Significance of the pQTL in the Icelandic Olink data
Effect_Amin_Iceland: Effect of the minor allele in the Icelandic Olink data
Same_Direction: Indicator if the direction of effect in the UKB-BI and Icelandic Olink data is concordant
Replicates: Indicator if the UKB-BI association is replicated in the Icelandic Olink data (P<0.05)

ST36: Replication of pQTLs detected in the Olink data (UKB-BI) in the SomaScan data (Iceland)

Chrom: Chromosome of the variant
Pos_IS: Position of the variant in the Icelandic data
Name_IS: Name of the variant in the Icelandic data
Pos_UK: Position of the variant in the UKB-BI data
Name_UK: Name of the variant in the UKB-BI data
pQTL_ID: pQTL ID in the UKB-BI data
cis_trans: Indicator if the pQTL is cis or trans for the protein
gene_prot: Name of the gene encoding the protein
SeqId: Internal SomaLogic assay ID
OlinkID: Internal Olink assay ID
rsName: RS name of the variant
close_genes: Genes close to the variant
Amin: Minor allele of the variant

Amaj: Major allele of the variant
Info_UK: Imputation info in the UKB-BI data set
MAF_PC_UK: Minor allele frequency of the variant in the UKB-BI data set (%)
Info_IS: Imputation info in the Icelandic data set
MAF_PC_IS: Minor allele frequency of the variant in the Icelandic data set (%)
P_UK: Significance of the association in the UKB-BI data set
Effect_Amin_UK: Effect of the minor allele in the UKB-BI data set
P_IS: Significance of the association in the Icelandic data set
Effect_Amin_IS: Effect of the minor allele in the Icelandic data set
same_direction: Indicator if the direction of effect is concordant between the UKB-BI and Icelandic data sets
P_repl_nom: The replication in the Icelandic data set is nominally significant
P_repl_bonf: The replication in the Icelandic data set reaches Bonferroni-corrected significance

ST37: Replication of pQTLs detected in the SomaScan data (Iceland) in the Olink data (UKB-BI)

See S21

ST38: Correlation between pQTLs and credible set variants for pQTLs detected using Olink in the UKB-BI data set

Chrom: Chromosome of the variant
Pos: Position of the variant
OlinkID: The internal Olink ID of the assay
Affected_Protein_Gene_name: Name of the gene encoding the associated protein
pQTL_ID: Global identifier of the pQTL
region_ID_prot: Protein-specific indicator of sentinel pQTL association
CisOrTrans: Indicator if the pQTL association is cis or trans
Marker: The variant associating with the protein levels
rsName: RS name of the variant
MAF_PC: Minor allele frequency of the variant (%)
Info: Imputation info of the variant
Amin: Minor allele of the variant
Amaj: Major allele of the variant
close_genes: Closest genes to the variant
Log10_pval_gc_cor_unadj: Significance of the association without adjusting for other signals at the locus. Negative log10 of the p-value.
Log10_pval_gc_cor_adj: Significance of the association, adjusted for higher-rank signals at the locus. Negative log10 of the p-value.
Effect_Amin_unadj: Effect of the minor allele of the variant without adjusting for other signals at the locus.
Effect_Amin_adj: Effect of the minor allele of the variant, adjusted for higher-rank signals at the locus.
RankWithinLocus: Rank in step-wise conditional analysis, where sentinel variants have rank=1 and secondary variants rank 2, 3, and so on

NumberWithinLocus: Number of significant variants in step-wise conditional analysis
SentinelMarker: The variant at the locus with the most significant association with the protein
Affected_Protein_Gene_chrom: Chromosome of the gene encoding the protein
Affected_Protein_Gene_chromstart: Start of the gene
Affected_Protein_Gene_chromend: End of the gene
Strand: Strand of the gene
cis_eQTL_genes: Genes that harbor an eQTL variant in high LD with pQTL variant
PAV_genes: Genes that harbor a protein-altering (coding) variant in high LD with pQTL variant
UniProt: UniProt ID of the protein
r2cor_est: Estimated correlation between a) r^2 between the sentinel pQTL and other variants in the credible set, calculated in the UKB dataset and b) r^2 between the sentinel pQTL and other variants in the credible set, calculated in the Icelandic dataset
r2cor_l95: Lower limit of 95% confidence interval corresponding to r2cor_est.
r2cor_u95: Upper limit of 95% confidence interval corresponding to r2cor_est.
r2_IS_min: Minimum r^2 between the sentinel pQTL and other variants in the credible set, calculated in the Icelandic dataset
r2_IS_median: Median r^2 between the sentinel pQTL and other variants in the credible set, calculated in the Icelandic dataset
r2_IS_mean: Mean r^2 between the sentinel pQTL and other variants in the credible set, calculated in the Icelandic dataset
r2_IS_max: Maximum r^2 between the sentinel pQTL and other variants in the credible set, calculated in the Icelandic dataset
r2_UK_min: Minimum r^2 between the sentinel pQTL and other variants in the credible set, calculated in the UKB dataset
r2_UK_median: Median r^2 between the sentinel pQTL and other variants in the credible set, calculated in the UKB dataset
r2_UK_mean: Mean r^2 between the sentinel pQTL and other variants in the credible set, calculated in the UKB dataset
r2_UK_max: Maximum r^2 between the sentinel pQTL and other variants in the credible set, calculated in the UKB dataset
n_r2: Number of pairs of r^2 values used in the calculation of the estimated correlation in r2cor_est

ST39: Correlation between pQTLs and credible set variants for pQTLs detected using SomaScan in the Icelandic data set

Chrom: Chromosome of the variant
Pos: Position of the variant
Marker: The variant associating with the protein levels
SeqId: The SomaLogic internal ID of the assay
locusID_global: Global identifier of the pQTL
locusID_prot: Protein-specific indicator of sentinel pQTL association
gene_prot: Name of the gene encoding the associated protein
chr_prot: Chromosome of the gene encoding the protein
strand_prot: Strand of the gene
TSS_prot: Start of the gene transcription site

shortname_prot: Name of the protein
longname_prot: Full name of the protein
UniProt: UniProt ID of the protein
Variant: RS name of the variant (if applicable)
LD_class_size: Number of variants in high LD with the variant
LD_class: Variants in high LD with the variant
region_start
region_end
Amin: Minor allele of the variant
Amaj: Major allele of the variant
Info: Imputation info of the variant
MAF_PC: Minor allele frequency of the variant (%)
cis_trans: Indicator if the pQTL association is in cis or trans
closest_genes: Closest genes to the variant
cis_eqtl_coding_genes: Genes that harbor an eQTL variant in high LD with pQTL variant
beta_adj: Effect of the minor allele of the variant, adjusted for higher-rank signals at the locus
beta_unadj: Effect of the minor allele of the variant without adjusting for other signals at the locus
mLog10pval_adj: Significance of the association, adjusted for higher-rank signals at the locus. Negative log10 of the p-value
mLog10pval_unadj: Significance of the association without adjusting for other signals at the locus. Negative log10 of the p-value
rank_locus
count_locus
count_cis_eQTL_same_gene
prop_same_direction_cis_eqtl_same_gene
count_cis_eQTL_diff_gene
any_coding_same_gene
any_coding_diff_gene
Rank_Proteins_per_Locus
Count_Proteins_per_Locus
Rank_Loci_per_Protein
Count_Loci_per_Protein
Reported
Reported_rsid
Reported_P
Reported_PMID
Reported_r2
r2cor_est: Estimated correlation between a) r^2 between the sentinel pQTL and other variants in the credible set, calculated in the UKB dataset and b) r^2 between the sentinel pQTL and other variants in the credible set, calculated in the Icelandic dataset
r2cor_l95: Lower limit of 95% confidence interval corresponding to r2cor_est.
r2cor_u95: Upper limit of 95% confidence interval corresponding to r2cor_est.
r2_IS_min: Minimum r^2 between the sentinel pQTL and other variants in the credible set, calculated in the Icelandic dataset

r2_IS_median: Median r^2 between the sentinel pQTL and other variants in the credible set, calculated in the Icelandic dataset

r2_IS_mean: Mean r^2 between the sentinel pQTL and other variants in the credible set, calculated in the Icelandic dataset

r2_IS_max: Maximum r^2 between the sentinel pQTL and other variants in the credible set, calculated in the Icelandic dataset

r2_UK_min: Minimum r^2 between the sentinel pQTL and other variants in the credible set, calculated in the UKB dataset

r2_UK_median: Median r^2 between the sentinel pQTL and other variants in the credible set, calculated in the UKB dataset

r2_UK_mean: Mean r^2 between the sentinel pQTL and other variants in the credible set, calculated in the UKB dataset

r2_UK_max: Maximum r^2 between the sentinel pQTL and other variants in the credible set, calculated in the UKB dataset

n_r2: Number of pairs of r^2 values used in the calculation of the estimated correlation in r2cor_est
