# Supplementary material for: Large-scale plasma proteomics comparisons through genetics and disease associations
Source: Nature. 2023 Oct 4;622(7982):348–58. doi: 10.1038/s41586-023-06563-x (PMC10567571; doi:10.1038/s41586-023-06563-x)
Supplement: Supplementary file 2 — Reporting Summary [file 41586_2023_6563_MOESM2_ESM.pdf]

nature portfolio

Corresponding author(s): Patrick Sulem, Kari Stefansson

Last updated by author(s): Aug 16, 2023

Reporting Summary

Nature Portfolio wishes to improve the reproducibility of the work that we publish. This form provides structure for consistency and transparency in reporting. For further information on Nature Portfolio policies, see our Editorial Policies and the Editorial Policy Checklist.

Statistics

For all statistical analyses, confirm that the following items are present in the figure legend, table legend, main text, or Methods section.

n/a	Confirmed	
☐	☒	The exact sample size (*n*) for each experimental group/condition, given as a discrete number and unit of measurement
☐	☒	A statement on whether measurements were taken from distinct samples or whether the same sample was measured repeatedly
☐	☒	The statistical test(s) used AND whether they are one- or two-sided<br>*Only common tests should be described solely by name; describe more complex techniques in the Methods section.*
☐	☒	A description of all covariates tested
☐	☒	A description of any assumptions or corrections, such as tests of normality and adjustment for multiple comparisons
☐	☒	A full description of the statistical parameters including central tendency (e.g. means) or other basic estimates (e.g. regression coefficient) AND variation (e.g. standard deviation) or associated estimates of uncertainty (e.g. confidence intervals)
☐	☒	For null hypothesis testing, the test statistic (e.g. *F*, *t*, *r*) with confidence intervals, effect sizes, degrees of freedom and *P* value noted<br>*Give P values as exact values whenever suitable.*
☒	☐	For Bayesian analysis, information on the choice of priors and Markov chain Monte Carlo settings
☒	☐	For hierarchical and complex designs, identification of the appropriate level for tests and full reporting of outcomes
☐	☒	Estimates of effect sizes (e.g. Cohen's *d*, Pearson's *r*), indicating how they were calculated

Our web collection on statistics for biologists contains articles on many of the points above.

Software and code

Policy information about availability of computer code

Data collection

We used the following publicly available software for data collection in the sequence processing pipeline:
BamQC (v1.0.0, https://github.com/DecodeGenetics/BamQC),
RTG Tools (v3.8.4, https://github.com/RealTimeGenomics/rtg-tools),
bcl2fastq (v2.20.0.422, https://support.illumina.com/sequencing/sequencing_software/bcl2fastq-conversion-software.html),

To process data generated on the Olink platform:
Olink Explore (v1.9.0, https://www.olink.com/products-services/data-analysis-products/npx-explore/)

Data analysis

We used the following publicly available software in conjunction with the algorithms described above.

GraphTyper (v2.7.1, v1.4, v2.7.2, https://github.com/DecodeGenetics/graphtyper),
GATK resource bundle (v4.0.12, gs://genomics-public-data/resources/broad/hg38/v0),
Svimmer (v0.1, https://github.com/DecodeGenetics/svimmer),
popSTR (v2.0, https://github.com/DecodeGenetics/popSTR),
Admixture (v1.3.0, https://dalexander.github.io/admixture),
Dipcall (v0.1, https://github.com/lh3/dipcall),
Samtools (v1.9, v1.3, https://github.com/samtools/samtools),
samblaster (v0.1.24, https://github.com/GregoryFaust/samblaster),
BWA (v0.7.10 mem, https://github.com/lh3/bwa),

GenomeAnalysisTKLite (v2.3.9, https://github.com/broadgsa/gatk),
Picard tools (v1.117, https://broadinstitute.github.io/picard),
Bedtools (v2.25.0-76-g5e7c696z, https://github.com/arq5x/bedtools2),
Variant Effect Predictor (release 100, https://github.com/Ensembl/ensembl-vep),
BOLT-LMM (v2.1, https://data.broadinstitute.org/alkesgroup/BOLT-LMM/downloads),
IMPUTE2 (v2.3.1, https://mathgen.stats.ox.ac.uk/impute/impute_v2.html),
dbSNP (v140, https://www.ncbi.nlm.nih.gov/SNP),
BiNGO (v3.0.3, https://www.psb.ugent.be/cbd/papers/BiNGO/Download.html),
Cytoscape (v3.7.1, https://cytoscape.org/download.html),
COLOC (v5.1.0.1, https://github.com/chr1swallace/coloc).

Data was analyzed and figures generated using Python (version 3.9.1), along with packages numpy (version 1.20.3), scipy (version 1.7.1), matplotlib (version 3.4.3), and pandas (version 1.3.0), and R (version 3.6.0).

The code central to power analysis and calculation of CV ratio can be found at https://github.com/DecodeGenetics/proteomics_comparison

For manuscripts utilizing custom algorithms or software that are central to the research but not yet described in published literature, software must be made available to editors and reviewers. We strongly encourage code deposition in a community repository (e.g. GitHub). See the Nature Portfolio guidelines for submitting code & software for further information.

Data

Policy information about availability of data

All manuscripts must include a data availability statement. This statement should provide the following information, where applicable:

- Accession codes, unique identifiers, or web links for publicly available datasets
- A description of any restrictions on data availability
- For clinical datasets or third party data, please ensure that the statement adheres to our policy

WGS, genotype data, phased and imputed data for the UK Biobank data set, as well as the proteomics data, can be accessed via the UKB research analysis platform (RAP), https://ukbiobank.dnanexus.com/landing. The UK Biobank Resource was used under application number 65851.
The Icelandic genomic data and proteomics data have been described in our previous publication2. While these individual-level data cannot be shared as dictated by the Icelandic law, we are open to collaborations on these topics, as we have been in the past.

GWAS summary statistics for all 2,931 Olink assays and all 4,907 SomaScan assays are available at https://www.decode.com/summarydata/.

Other data presented in this study are included in this publication (and its Supplementary Information).

URLs for other external data used are as follows:
the GWAS Catalog (https://www.ebi.ac.uk/gwas/),
the GTEx project (https://gtexportal.org/home/),
the Human Protein Atlas (https://www.proteinatlas.org/),
STRING database (https://string-db.org/, file name: 9606.protein.actions.v11.txt.gz),
UniProt (https://www.uniprot.org/).

Research involving human participants, their data, or biological material

Policy information about studies with human participants or human data. See also policy information about sex, gender (identity/presentation), and sexual orientation and race, ethnicity and racism.

Reporting on sex and gender	Self-reported sex of subjects was recorded at enrollment. Statistical analyses are adjusted for sex based on self-reported sex.
Reporting on race, ethnicity, or other socially relevant groupings	The UK Biobank data set is stratified by ancestry into subjects of British and Irish, South-Asian, and African ancestry, based on genomic information as described in Halldorsson, B. V. et al. The sequences of 150,119 genomes in the UK Biobank. Nature 607, 732–740 (2022).
Population characteristics	The population characteristics of the Icelandic data set have been described in Ferkingstad, E. et al. Large-scale integration of the plasma proteome with genetics and disease. Nat. Genet. 53, 1712–1721 (2021).<br>The population characteristics of the UK Biobank have been described in Sudlow, C. et al. UK Biobank: An Open Access Resource for Identifying the Causes of a Wide Range of Complex Diseases of Middle and Old Age. PLOS Med. 12, e1001779 (2015).
Recruitment	See 'Population characteristics'.
Ethics oversight	All participants who donated samples to the Icelandic proteomics data set gave informed consent. The study was approved by the National Bioethics Committee of Iceland and conducted in agreement with conditions issued by the Data Protection Authority of Iceland (VSN_14-015). Personal identities of the participants were encrypted by a third-party system (Identity Protection System) approved and monitored by the Data Protection Authority.<br>The scientific protocol and operational procedures of the UK Biobank were reviewed and approved by the North West

Research Ethics Committee (REC Reference Number: 06/MRE08/65). Data for this study were obtained and research conducted under UKB application license numbers 24898, 68574 and 65851

Note that full information on the approval of the study protocol must also be provided in the manuscript.

Field-specific reporting

Please select the one below that is the best fit for your research. If you are not sure, read the appropriate sections before making your selection.

☒ Life sciences ☐ Behavioural & social sciences ☐ Ecological, evolutionary & environmental sciences

For a reference copy of the document with all sections, see nature.com/documents/nr-reporting-summary-flat.pdf

Life sciences study design

All studies must disclose on these points even when the disclosure is negative.

Sample size	In Iceland, all individuals with available plasma samples have been analyzed by Somascan proteomics platform. The sample size in Iceland is comparable to the one used in the UK with Olink, where there majority of assays have cis pQTL association. Samples for measurement using the Olink platform in the Icelandic data set were chosen pseudo-randomly from those previously measured using the SomaScan platform. No statistical analysis was performed to choose sample size for that subset.
Data exclusions	We had two preestablished exclusion criteria. First, Non-human proteins were excluded because the aim of this study is to assess human biology. Second, deprecated assays according to the manufacturers of the Olink and SomaScan assays were excluded from the analysis. These assays are excluded from the beginning and are not taken into account in the counts. Assays mapping to multiple genes were excluded from the Somascan platform since when multiple proteins can be measured at the same time, it is difficult to classify associations as cis or trans. Individuals with evidence of incorrect labelling based on the comparison of their protein levels with genotypes and phenotypes were excluded from all the analyses.
Replication	We attempted replication in two different ways: a) between Olink and Somascan platforms and b) within Olink platform. We attempted to replicate pQTLs detected in the British and Irish subset of the UK Biobank data set using the Olink platform in Icelandic data set using the SomaScan platform and vice versa. We attempted to replicate the associations detected in UK Biobank using Olink on a subset of individuals with available plasma proteomics in Iceland also using Olink.
Randomization	We performed an observational study where we test association between protein level and sequence variants or phenotypes using two platforms and compare the results. There is no intervention in this study. The protein levels, genotypes and phenotypes are solely observed but never assigned as would be the case in an interventional study (e.g. clinical trial). Thus, randomization is not applicable to this study.
Blinding	The protein levels, genotypes and phenotypes were measured or assessed completely independently of each other.

Reporting for specific materials, systems and methods

We require information from authors about some types of materials, experimental systems and methods used in many studies. Here, indicate whether each material, system or method listed is relevant to your study. If you are not sure if a list item applies to your research, read the appropriate section before selecting a response.

Materials & experimental systems

n/a	Involved in the study	
☒	☐	Antibodies
☒	☐	Eukaryotic cell lines
☒	☐	Palaeontology and archaeology
☒	☐	Animals and other organisms
☒	☐	Clinical data
☒	☐	Dual use research of concern
☒	☐	Plants

Methods

n/a	Involved in the study	
☒	☐	ChIP-seq
☒	☐	Flow cytometry
☒	☐	MRI-based neuroimaging
